# Gill-Inspired Dual-Effect All-Biomass Aerogel Evaporator Overcoming Salt Accumulation for Sustainable Solar Desalination

**DOI:** 10.1007/s40820-026-02284-8

**Published:** 2026-07-13

**Authors:** Nuo Liu, Chunqing Niu, Xin Zhang, Deqian Yang, Weiyang Gu, Guangyu Zhang, Haifeng Zhang, Chunhong Zhu, Jian Shi

**Affiliations:** 1 Graduate School of Medicine, Science and Technology, Shinshu University, Tokida 3-15-1, Ueda, Nagano, 386-8567 Japan; 2 Institute for Fiber Engineering and Science (IFES), Shinshu University, Tokida 3-15-1, Ueda, Nagano, 386-8567 Japan; 3 Graduate School of Science and Technology, Shinshu University, Tokida 3-15-1, Ueda, Nagano, 386-8567 Japan; 4 College of Textile and Clothing, Nantong University, Nantong, 226019 Jiangsu People’s Republic of China; 5 Faculty of Textile Science and Technology, Shinshu University, Tokida 3-15-1, Ueda, Nagano, 386-8567 Japan

**Keywords:** Gill-inspired design, Dual-effect synergy, All-biomass aerogel, Salt-resilient solar evaporation, Eco-safe, Sustainable desalination

## Abstract

The global shortage of freshwater is becoming increasingly severe, necessitating the development of green and efficient water treatment technologies to address challenges in high-salinity environments. Inspired by the salt excretion mechanism of marine fish gills, a molecular and interfacial synergistic construction (MISC) strategy was applied to build carboxymethyl chitosan (CMCS) and sodium alginate (SA) all biomass aerogel evaporators with high strength and excellent salt resistance. At the molecular level, the abundant charged and hydrophilic groups endow the aerogel with strong ion-regulation and water-affinity capabilities, giving rise to two interfacial processes: Donnan-based ionic exclusion and a water-film effect that drives salt removal through interfacial flow. With this dual-effect synergy, the evaporator achieved a high evaporation rate of 2.88 kg m^−2^ h^−1^ under 1 sun and operated stably for 24 h in 20 wt% saline water without salt deposition. The interpenetrated network of rigid CMCS and flexible SA further provides excellent mechanical properties and structural stability. Importantly, the evaporator is entirely composed of natural biomass materials, allowing for low-cost scalable fabrication with good biosafety and biodegradability. Overall, this work establishes a gill-inspired dual-effect framework for overcoming salt accumulation and offers a sustainable and efficient route for solar-driven desalination under extreme salinity.

**Supplementary Information:**

The online version contains supplementary material available at 10.1007/s40820-026-02284-8.

## Highlights

- A fish gill-inspired molecular and interfacial synergistic construction strategy enables a dual-effect salt rejection framework for solar evaporation.
- Synergistic coupling of Donnan exclusion and water-film driven Marangoni flow sustains interfacial hydration and continuous salt removal.
- The all biomass carboxymethyl chitosan/sodium alginate aerogel achieved 2.88 kg m^-2^ h^-1^ evaporation and stable operation in 20 wt% brine without salt deposition.

**Supplementary Information:**

The online version contains supplementary material available at 10.1007/s40820-026-02284-8.

## Graphical abstract

**Figure:**
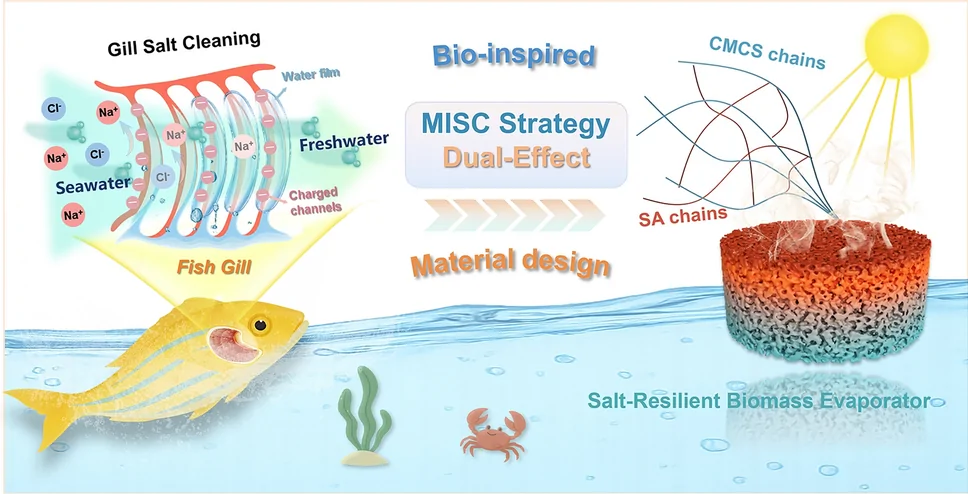

## Introduction

Freshwater is vital for human survival and sustainable development [1, 2]. However, only approximately 2.53% of the Earth’s total water is freshwater, and the share that can be directly accessed is even smaller [3]. This limited supply is facing increasing pressure as pollution and growing water demand continue to reduce the amount of clean water available [4]. Consequently, seawater, the most abundant water resource on the planet, is becoming an important pathway to alleviate global water stress [5]. Conventional desalination approaches, including reverse osmosis and multistage flash, are commercially viable but require high energy input, large infrastructure, and costly maintenance, which limits their use in remote or off-grid water-scarce areas [6]. Therefore, developing green, low-carbon, simple, and easy-to-deploy desalination systems has become an important research frontier at the intersection of energy and environment [7].

In recent years, solar-driven interfacial evaporation has attracted increasing attention as a promising approach for producing clean water. As a decentralized and renewable purification method, it further contributes to SDG 6 by advancing clean water accessibility and sustainable water resource management [8]. This technique features a simple device structure, efficient photothermal conversion, and strong adaptability to regions with limited infrastructure requirements. By localizing heat at the air–water interface rather than heating the entire water body, it significantly improves the efficiency of vapor generation [9–11].

However, when operated in high-salinity environments or during long-term desalination, interfacial evaporation systems inevitably suffer from salt crystallization [12–14]. During evaporation, continuous water loss induces an upward convective flux that transports the dissolved ions toward the evaporating interface. Because the diffusion of ions back into the bulk is relatively slow, a concentration-gradient boundary layer is formed near the surface [15]. Once the interfacial salinity exceeds the saturation point, heterogeneous nucleation occurs and salt rapidly crystallizes, producing a compact deposition layer on the evaporating surface [12, 16]. This salt layer severely inhibits light absorption, clogs interconnected pores, and disrupts capillary-driven water replenishment, ultimately leading to uneven interfacial wetting and local drying. These interfacial failures progressively increase the thermal resistance and sharply reduce the evaporation performance, eventually causing the complete operational failure of the evaporator [17, 18].

To mitigate salt crystallization, various salt-resistant strategies have been proposed; however, achieving long-term stable evaporation under high salinity remains a major challenge [19]. The core difficulty lies in the fact that evaporation continuously drives ions toward the interface, whereas water replenishment and ion back-diffusion cannot keep pace, leading to persistent interfacial supersaturation and repeated crystallization under harsh conditions. For example, crystallization–peeling strategies remove salt through mechanical detachment, but repeated peeling inevitably damages the material framework and cannot prevent recrystallization during continuous operation [20]. Janus structures suppress salt crystallization by spatially separating water and ion pathways; yet, their limited effective evaporation area results in relatively low overall efficiency [21]. In addition, the hydrophobic modification required for Janus behavior further restricts water transport, which can intensify interfacial salt accumulation and ultimately degrade performance [22]. Electrostatic or Donnan-type repulsion strategies alone perform well at low-to-moderate salinity; however, at high ionic strength, these charge-based interactions are strongly screened and thus fail to provide effective regulation of salt accumulation [17, 23]. Taken together, these strategies can partially reduce salt buildup but are unable to simultaneously achieve effective salt regulation and stable interfacial hydration under high-salinity and long-duration operations, making it difficult to maintain the long-term stability of the evaporating interface.

**Fig. 1:**
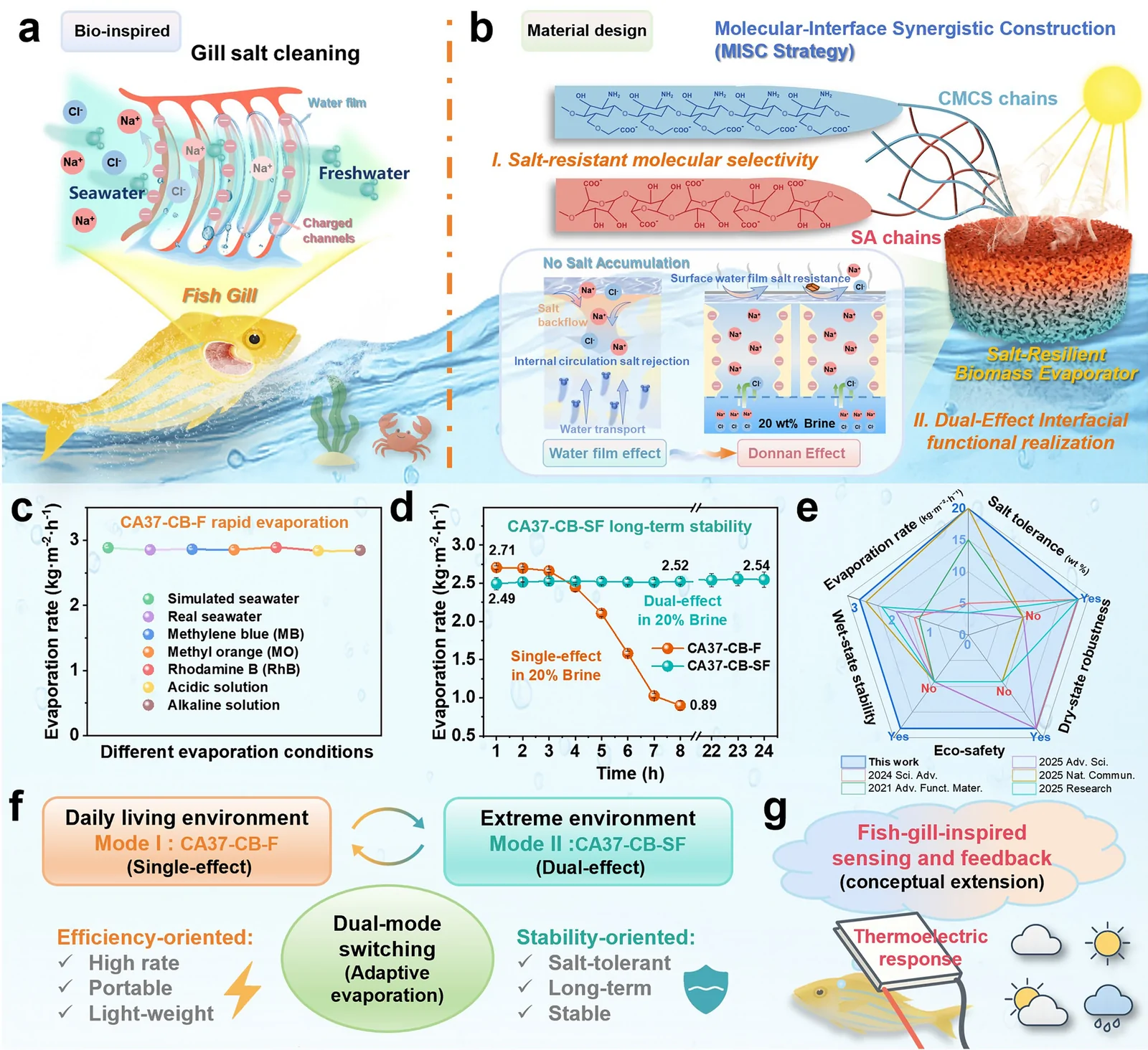
**a** Fish gill inspiration and **b** design of the salt-resilient biomass evaporator. **c** Rapid water evaporation of the CA37-CB-F evaporator under various daily environmental conditions in the single-effect mode. **d** Long-term stable evaporation of the CA37-CB-SF evaporator in highly concentrated brine (20 wt% NaCl) under the dual-effect mode. **e** Comparison of the CA37-CB evaporator with representative literature in terms of evaporation rate, salt tolerance, dry/wet stability, and eco-safety. **f** Application conditions and advantages of the dual-mode switching capability of the CA37-CB evaporator. **g** Conceptual extension toward thermoelectric sensing inspired by fish gill perception

Nature offers valuable inspiration for salt management. Marine fish can maintain osmotic balance in highly saline environments through evolutionarily optimized gills, which employ a coordinated mechanism for ion regulation and water transport. As illustrated in Fig. 1a, charged ion-regulating channels within the gill membranes selectively repel certain ions via Donnan-like electrostatic interactions [24, 25], while a thin, continuously hydrated water film ensures uninterrupted water transport and suppresses local salt accumulation [26–28]. This biologically derived dual-effect mechanism provides an effective conceptual framework for designing advanced materials for solar-driven interfacial evaporation.

Inspired by the dual-effect regulation of fish gills, we propose a molecular and interfacial synergistic construction (MISC) strategy to develop an integrated biomass aerogel evaporator with closely coupled material composition, microstructure, and interfacial functionality. As shown in Fig. 1b, carboxymethyl chitosan (CMCS) and sodium alginate (SA) are used as molecular building units. The introduction of charged functional groups (such as –COO^–^) at the molecular scale creates a gill-like ion repulsion layer at the evaporating interface and enables selective exclusion of anions (such as Cl^–^) through a Donnan-type mechanism. At the same time, the hydrophilic groups (such as –OH) distributed along the polymer chains maintain a continuous surface water film. Under photothermal excitation, this hydrated layer generates interfacial tension gradients and water-film convection that dynamically remove accumulated salts. Through this dual-effect synergy, which combines the Donnan effect and the water-film effect, the constructed salt-resilient biomass evaporator achieves effective and stable interfacial salt management. As illustrated in Figs. 1c, d, f, and 5a, the evaporator can be demonstrated in two operational modes, namely CA37-CB-F mode (dominated by the single Donnan effect) and the CA37-CB-SF mode (involved both Donnan and water-film effect). The CA37-CB-F mode delivers rapid evaporation with a rate of 2.88 kg m^−2^ h^−1^ in simulated seawater with a salinity of 3.5 wt% and maintains similar performance in real seawater and various contaminated water sources. In contrast, the CA37-CB-SF mode is designed for extreme salinity. It achieves uninterrupted operation for 24 h in brine containing 20 wt% NaCl solution without salt crystallization or performance decay, demonstrating strong salt resilience and long-term evaporation stability.

In addition, the interpenetrating network formed by the rigid CMCS backbone and the flexible SA segments provides compressive strength in the dry state and recoverability in the wet state. This structural robustness is essential for gill-inspired dual-effect interfacial regulation. The evaporator is constructed entirely from renewable natural polymers and exhibits low cost, environmental safety, and full biodegradability. In terms of evaporation rate, salt resilience, dry, and wet stability and eco-safety, the CA37-CB system outperformed representative photothermal evaporators reported in the literature [12, 29–32] (Fig. 1e). Moreover, as shown in Fig. 1g, the gill-inspired salt regulation concept may be further extended at a conceptual level to thermoelectric sensing for environmental monitoring during solar-driven operation [33, 34]. Overall, this work presents a bioinspired material-design concept that couples molecular level charge regulation with interfacial water-film dynamics. This integrated approach enables the intelligent control of interfacial salt accumulation and provides a promising pathway for efficient solar evaporation and sustainable water management under extreme salinity.

## Experimental Section

### Materials

Carboxymethyl chitosan (CMCS) was purchased from Shandong Xiya Chemical Co., Ltd. (China). Sodium alginate (SA), glutaraldehyde (GA, 25%), and anhydrous calcium chloride (CaCl_2_) were obtained from the FUJIFILM Wako Pure Chemical Corporation (Japan). The biomass-derived photothermal carbon black (CB) used in this work was prepared by high-temperature carbonization of bamboo. Sodium chloride (NaCl), methylene blue (MB), methyl orange (MO), rhodamine B (RhB), sulfuric acid (H_2_SO_4_), and sodium hydroxide (NaOH) used to prepare the simulated water sources were also supplied by FUJIFILM Wako Pure Chemical Corporation (Japan). Natural seawater was collected from Yurihonjo, Akita, Japan (39°23′39.2″N, 140°00′40.4″E).

### Preparation of Biomass-based CA37-CB Aerogel Evaporator

CMCS and SA powders were separately dissolved in deionized water and stirred in a 70 °C water bath to prepare 2 wt% CMCS and 2 wt% SA solutions. CB powder (0.12 g) was added to 1 mL of deionized water and dispersed by ultrasonication, followed by the addition of 3 mL of CMCS solution and 7 mL of SA solution. The mixture was stirred thoroughly to obtain a uniform black slurry. Subsequently, 1 mL of a 2 wt% glutaraldehyde solution was added to the slurry, and the mixture was quickly transferred into a homemade mold (diameter 3 cm and height 1 cm) to initiate the preliminary crosslinking of CMCS and form a hydrogel. The hydrogel was frozen at − 20 °C for 12 h and then freeze-dried for 24 h. The dried sample was immersed in a 1 wt% CaCl_2_ solution to further crosslink the SA network and then soaked in deionized water for 24 h to remove excess calcium ions. After a final drying step, the biomass CA37-CB aerogel evaporator was prepared. The control samples CA37 (without CB), CA19-CB (CMCS/SA = 1/9), CA55-CB (CMCS/SA = 5/5), CA73-CB (CMCS/SA = 7/3), and CA91-CB (CMCS/SA = 9/1) were prepared using the same procedure.

### Solar-Driven Interfacial Evaporation Experiment

Solar-driven interfacial evaporation experiments of the biomass aerogels were performed out using a homemade evaporation system. First, 100 mL of the test solution (according to the experimental requirements) was placed in a 100 mL beaker. The evaporator was operated in two modes in this work (Fig. 5a) to evaluate the daily rapid evaporation performance (Mode I: CA37-CB-F) and long-term stability in high-salinity brine (Mode II: CA37-CB-SF). For the CA37-CB-F mode, the aerogel evaporator was placed on polystyrene insulation foam and connected to the test solution through hydrophilic foam to ensure continuous water supply. For the CA37-CB-SF mode, the aerogel was placed at the center of the polystyrene insulation foam and directly contacted the test solution to achieve a self-floating water supply. AG-CB-SF was also tested under the same Mode II configuration as a water film-only control, in which near-neutral agar (AG) replaced the charged CMCS/SA network. The full characterization procedures and details of the solar-driven interfacial evaporation experiments are provided in Note S1.

The evaporation rate (v) was calculated using Eq. (1) [35].

1 $$v = \frac{\Delta m}{{S \times \Delta t}}$$

where v represents the evaporation rate, Δm is the water evaporation mass change, S represents the area of the evaporation interface, and Δt refers to the solar illumination time.

A dark evaporation experiment was designed to estimate the equivalent evaporation enthalpy ($\Delta {H}_{e}$) for different samples, which was calculated by Eq. (2) [36].

2 $$U_{in} = \Delta H_{w} m_{w} = \Delta H_{e} m_{e}$$

where $\Delta {H}_{w}$ is the known theoretical value of 2393 J g^−1^ of liquid water, ${m}_{\mathrm{w}}$ and ${m}_{e}$ are the mass change of water and aerogel samples.

The solar steam evaporation efficiency (*η*) is defined as Eqs. (3)–(5) [37, 38].

3 $$\eta = \frac{{\dot{m} \left( {H_{pc} + H_{s} } \right)}}{{C_{opt} }}$$

4 $$H_{pc} = 1.91846 \times 10^{6} \times \left[ {T_{1} /\left( {T_{1} - 33.91} \right)} \right]^{2}$$

5 $$H_{s} = C \times \left( {T_{1} - T_{0} } \right)$$

where η represents the steam evaporation efficiency, $\dot{m}$ denotes the net water evaporation rate under the corresponding solar illumination intensity (deducting the rate of evaporation under dark conditions), ${H}_{pc}$ is the latent heat of water from the liquid to vapor state, ${H}_{s}$ is the sensible heat of water by increasing the water temperature, ${C}_{opt}$ is the simulated solar intensity on the top surface of the evaporator, C is the specific heat capacity of water (4.18 kJ kg^–1^ K^–1^), and ${T}_{0}$ and ${T}_{1}$ are the initial water temperature and evaporation surface temperature, respectively.

## Results and Discussion

### Fabrication and Characteristics of the Solar Evaporation System

**Fig. 2:**
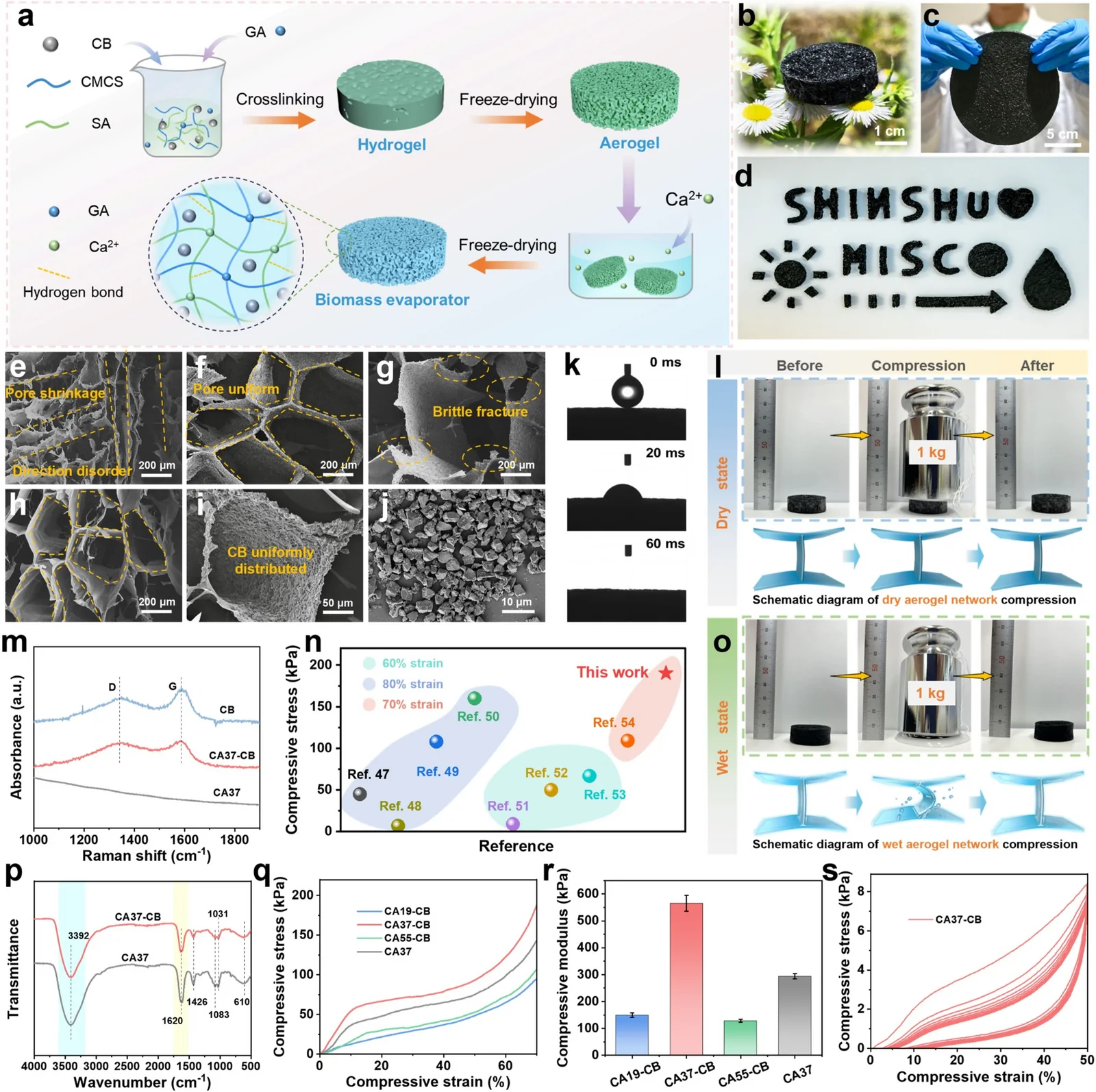
**a** Schematic of the fabrication process of the fish gill-inspired biomass aerogel evaporator. **b** Display of the ultralight property of the aerogel. **c** Scalable fabrication of the aerogel. **d** Shape adaptability of the aerogel. SEM images of aerogels with different CMCS/SA ratios and bamboo charcoal (CB): **e** CA19-CB, **f**, **i** CA37-CB, **g** CA55-CB, **h** CA37, and **j** CB. **k** Wettability of CA37-CB aerogel. Pressure test with heavy load: **l** dry rigidity and **o** wet compression recovery. **m** Raman spectra of CB and CA37-CB aerogel. **n** Comparison of compressive stress at 70% strain with reported aerogels. **p** FTIR spectrum of CA37-CB aerogel. **q** Dry state compressive stress–strain curve. **r** Dry state compressive modulus. **s** Wet state cyclic compression test

Inspired by fish gills, biomass aerogel evaporators with strong hydrophilicity, high mechanical performance, and salt resistance were designed and fabricated using CMCS and SA as the main materials (Fig. 2a). CMCS and SA were dissolved in water with CB as the natural photothermal material. After adding GA as a crosslinker, a semi-interpenetrating hydrogel was obtained. The hydrogel was freeze-dried into an aerogel and then immersed in a Ca^2+^ solution to crosslink SA, resulting in the CMCS/SA-CB aerogel evaporator.

To obtain a stable structure, strong mechanical performance, and high evaporation efficiency, aerogels with different CMCS/SA ratios were prepared and their morphologies were observed using SEM. As shown in Fig. 2e–g, the CA19-CB aerogel contained more flexible SA chains [39], resulting in small and disordered pores. The blocked channels hindered water transport. With a more rigid CMCS [40], the CA37-CB aerogel formed a regular interconnected porous network. This facilitated rapid water transport to the evaporation surface and allowed rapid vapor diffusion, which reduced vapor pressure and promoted continuous evaporation [41]. With even higher CMCS, the CA55-CB aerogel became brittle and unstable, and cracks appeared in the skeleton, blocking the continuous water supply. When the CMCS content was further increased, CA73-CB and CA91-CB aerogels could not be formed (Fig. S1). As shown in Fig. 2h–j, the CA37 aerogel without CB also had a uniform porous structure. CB had a particle size of about 3 µm. After incorporation into CA37 aerogel, CB was distributed uniformly inside and partially exposed on the pore walls, which increased the surface area and improved light-to-heat conversion.

Figure 2b shows the ultralight feature of the CA37-CB aerogel. With its highly porous structure, the aerogel weighed only 0.3 g and could rest on a flower without being deformed. In practical desalination scenarios, large-area evaporators are required to meet the actual water demand. Therefore, aerogels with diameters of up to 15 cm were fabricated (Fig. 2c), demonstrating the scalability of the material system. As shown in Fig. 2d, the CA37-CB aerogel can be molded into different shapes, indicating excellent shape adaptability and suitability for various deployment conditions. This geometric adaptability also supports the broader goal of the MISC strategy, which aims to enable practical, sustainable, and scenario-flexible solar desalination. Figure 2k shows the rapid wetting and water absorption behavior of the CA37-CB aerogel. A water droplet spread and was absorbed within 60 ms, indicating strong hydrophilicity and rapid interfacial water transport. Such strong hydrophilicity is essential for forming a stable surface water film under illumination, which facilitates the water film effect and accelerates water replenishment at the evaporating interface. Because all the components were hydrophilic biomass, the aerogels also exhibited high water uptake (Fig. S2). CA37-CB absorbed water up to 1564%, enabling sufficient water storage and a continuous water supply during evaporation. This high water-holding capacity supports persistent water film formation and stable evaporation, even in high-salinity brine.

In Fig. 2m, CB showed two characteristic peaks at about 1350 cm^−1^ (D band) and 1580 cm^−1^ (G band). The stronger G band indicated a relatively high degree of graphitization, confirming the successful preparation of photothermal CB [42]. This graphitized structure is beneficial for efficient solar absorption and heat localization. After incorporation, CA37-CB aerogel exhibited the same D and G bands, demonstrating the uniform loading of CB within the aerogel matrix. FTIR analysis (Fig. 2p) was conducted to verify the chemical composition, crosslinking structure, and functional groups responsible for interfacial ion regulation. Both CA37 and CA37-CB aerogels contained abundant –COO^–^, –OH, and –NH₂ groups originating from CMCS and SA. Notably, the –COO^–^ groups provide fixed negative charges that can repel co-ions (e.g., Cl^–^) through a Donnan-type mechanism, while the polar –OH and –NH₂ groups enhance hydrophilicity and support the formation of a stable water film. The broad band at 3200–3500 cm^−1^ corresponded to O–H stretching, confirming the presence of hydroxyl-rich surfaces [43]. The peak near 1620 cm^−1^ was attributed to C = N stretching from the Schiff-base reaction between GA and CMCS amino groups, indicating the formation of the CMCS network [44]. The symmetric stretching vibration of –COO^–^ at 1426 cm^−1^ originated from SA [45], and the weak peaks at 500–700 cm^−1^ arose from Ca^2+^–carboxylate coordination, demonstrating that SA underwent effective ionic crosslinking [46]. Peaks at 1083 and 1031 cm^−1^ (C–O and C–O–C vibrations) showed that the polysaccharide backbones remained intact. Collectively, these results confirm that CA37-CB forms a robust dual-network structure, consisting of a covalently crosslinked CMCS framework and an ionically crosslinked SA framework, while simultaneously providing ion-repelling and water-binding functional groups. This molecular architecture underpins the dual-effect interfacial regulation (Donnan effect and water film effect) and enables rapid water evaporation as well as long-term salt-resistant performance under high salinity.

XPS analysis was further performed to verify the surface chemical composition and bonding environment of the CA37-CB aerogel (Figs. S3 and S4). The survey spectrum confirmed the presence of C, O, N, and Ca elements, consistent with the CMCS/SA-based dual-network structure. In the high-resolution C 1*s* spectrum, the C–O/C–N component at 286.54 eV, together with the N 1*s* signal at 399.88 eV, confirms the presence of CMCS-related nitrogen-containing groups and supports the Schiff-base crosslinking between glutaraldehyde and CMCS. Meanwhile, the O–C = O component in C 1*s* at 288.18 eV and the C = O/COO⁻ component in O 1*s* at 531.38 eV verify the abundant carboxylate-containing groups derived from SA and CMCS, which provide fixed anionic sites for Donnan-type ion exclusion. In addition, the characteristic Ca 2*p* doublet at 347.58 and 351.08 eV confirms Ca^2+^–carboxylate coordination in the SA network. These results agree well with the FTIR analysis, demonstrating the successful formation of a covalently crosslinked CMCS network and an ionically crosslinked SA network, while also confirming the hydrophilic and negatively charged chemical environment required for dual-effect salt regulation. With rigid CMCS and flexible SA, CA37-CB aerogel provided sufficient mechanical robustness to support long-term evaporation. As shown in Fig. 2l, q and r, a 1 kg load placed on a 0.3 g aerogel did not cause visible deformation, indicating excellent load-bearing capacity in the dry state. In uniaxial compression, CA37-CB exhibited a stress of 190 kPa at 70% strain and a modulus of 550 kPa, which were much higher than those of CA19-CB, CA55-CB and many reported biomass aerogels (Fig. 2n) [47–54]. This level of stiffness ensures that the evaporator can maintain its macroscopic shape and structural integrity during handling and operation. The enhanced dry strength mainly originated from the rigid CMCS-based skeleton reinforced by Schiff-base crosslinking, while the incorporation of flexible SA chains reduced brittleness and endowed the network with a certain degree of toughness. Mechanical durability in the wet state is also important for stable operation under solar evaporation. As shown in Fig. 2o, s, CA37-CB aerogel showed good recovery after wet compression. Under a 1 kg load, water inside the aerogel was squeezed out, but the structure rapidly recovered its original shape after unloading. After 10 cycles of wet compression, the aerogel still maintained a compressive strength of about 8.39 kPa without obvious structural damage. This behavior can be attributed to the combination of high water uptake and the cooperative support of the CMCS rigid framework and SA flexible segments. Therefore, CA37-CB aerogel, as a biomass-based evaporator, possesses adequate mechanical strength and stability to withstand repeated loading and long-term solar-driven evaporation.

### Photothermal Conversion and Water Evaporation

**Fig. 3:**
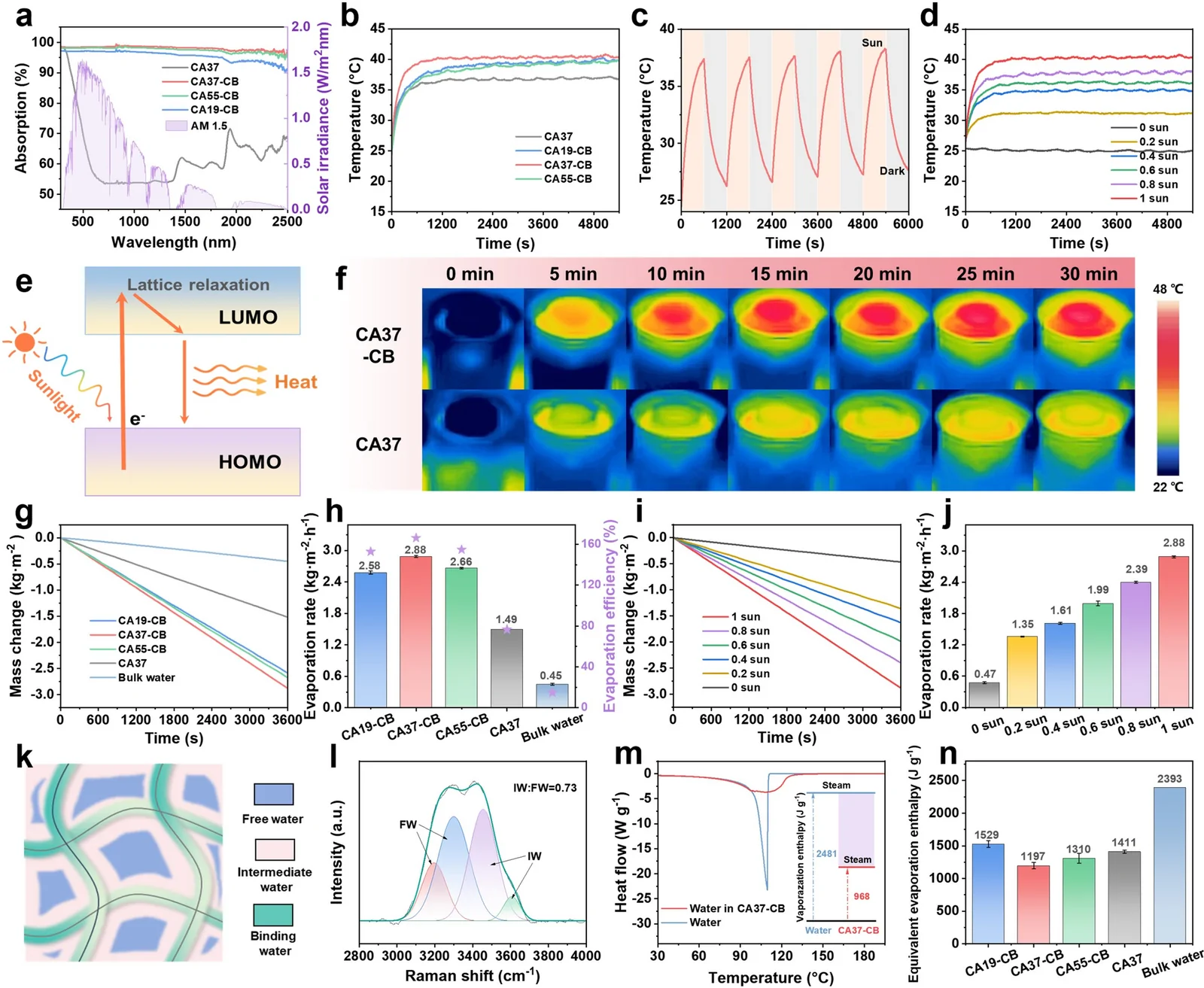
**a** Solar absorption spectra of fish gill-inspired biomass aerogels. **b** Surface temperature of biomass aerogel evaporators with different ratios under 1 sun. **c** Photothermal conversion stability of CA37-CB biomass aerogel evaporator. **d** Surface temperature of CA37-CB evaporator under different light intensities. **e** Photothermal conversion mechanism of CA37-CB evaporator.** f** Infrared thermal images of the CA37 and CA37-CB evaporators under sunlight. **g** Mass loss of biomass aerogel evaporators with different CMCS/SA ratios under 1 sun. **h** Evaporation rate and efficiency of biomass aerogel evaporators under 1 sun. **i**, **j** Mass loss and evaporation rate of CA37-CB evaporator under different light intensities. **k** Water states in CA37-CB aerogel network. **l** Raman spectra and fitting peaks of water in CA37-CB aerogel. **m** DSC curves and evaporation enthalpy of water in CA37-CB aerogel. **n** Comparison of equivalent evaporation enthalpy

The fish gill-inspired biomass aerogel evaporator exhibited excellent hydrophilicity and mechanical robustness, as well as strong light absorption and photothermal conversion capabilities. As shown in Fig. 3a, the CA37 aerogel without CB already shows more than 50% solar absorption across the solar spectrum, which originates from its hierarchical pore structure and large specific surface area [55]. Biomass-derived carbon black (CB) further enhances optical absorption owing to its broadband light-harvesting ability and the additional surface roughness introduced upon incorporation, as supported by the SEM observation of CA37-CB (Fig. 2f, i). Consequently, the CA37-CB aerogel achieves an ultra-high absorption of up to 98% over the entire solar spectrum. The photothermal performance of CA37-CB was evaluated under 1 sun illumination. As shown in Fig. 3b, the aerogel rapidly converted the absorbed sunlight into heat, and the surface temperature increased sharply during the first 10 min, eventually stabilizing at approximately 40 °C after 20 min. This efficient temperature increase is attributed to the interconnected porous structure and uniform distribution of CB. As a carbon-based photothermal material, CB absorbs solar energy over a broad wavelength range, and the excited electrons dissipate energy through non-radiative relaxation to generate localized heat. The heat is then transferred through the aerogel network via thermal conduction and interfacial heat transfer, enabling rapid and stable photothermal conversion [56] (Fig. 3e).

To evaluate the photothermal stability of the CA37-CB biomass aerogel evaporator, light–dark cycling tests were conducted [57] (Fig. 3c). CA37-CB exhibited an immediate thermal response upon illumination, and the surface temperature increased rapidly within the first 10 min. Once the light was turned off, the temperature quickly dropped back to the baseline, confirming its reversible and stable photothermal behavior. As shown in Fig. 3d, the photothermal performance under weak light was further examined to simulate practical scenarios such as cloudy weather. The evaporator remained at room temperature in the dark. When the illumination intensity increased, the surface temperature rose accordingly. Even under 0.4 sun, the temperature increased by approximately 10 °C, demonstrating that the evaporator can operate efficiently under low-intensity light. The corresponding thermal images in Fig. 3f directly display the heating processes of the CA37 and CA37-CB aerogels. The heat generated by photothermal conversion is concentrated locally inside the aerogel, rather than spreading into the bulk water. This improves the energy utilization efficiency and ensures efficient photothermal water evaporation [58, 59].

Based on the strong photothermal conversion capability of the biomass aerogel evaporator, its evaporation performance under simulated sunlight was further examined to identify its optimum composition. As shown in Fig. 3g, h, bulk water exhibits an evaporation rate of only 0.45 kg m^−2^ h^−1^. Even the CA37 aerogel without CB achieves 1.49 kg m^−2^ h^−1^ owing to its uniform pore structure and good thermal localization. Consistent with the photothermal results, the CA37-CB evaporator exhibited the best performance among all the compositions. Compared with CA19-CB and CA55-CB, CA37-CB exhibits a more interconnected and homogeneous porous network, more uniform dispersion of CB, and more efficient interfacial heat confinement [60]. The evaporation rate and apparent solar-to-vapor efficiency reached 2.88 kg m^−2^ h^−1^ and 166.14%, respectively (Note S4). To further evaluate the feasibility of the evaporator under weak illumination, the light intensity was gradually reduced. As shown in Fig. 3i, j, the CA37-CB aerogel maintained evaporation rates of 0.47, 1.35, 1.61, 1.99, and 2.39 kg m^−2^ h^−1^ at 0, 0.2, 0.4, 0.6, and 0.8 kW m^−2^, respectively. Compared with bulk water (0.45 kg m^−2^ h^−1^), the enhancement remained significant even at 0.4 sun, demonstrating reliable operation under cloudy or low-light conditions. Importantly, the CA37 ratio was selected as the optimal formulation based on its balanced pore morphology, superior mechanical integrity, and highest evaporation performance. Therefore, subsequent mechanistic analyses were performed using the optimized CA37-CB aerogel.

As shown in Fig. 3k, according to the differences in hydrogen bonding between water molecules and surrounding molecules, water in the polymer network contains three distinct states: free water (FW, blue), intermediate water (IW, pink), and bound water (BW, green) [61]. The above experiments demonstrate that the CA37-CB aerogel evaporator exhibits high evaporation rate because the evaporation enthalpy decreases due to water activation [62]. To investigate the distribution of different types of water in the CA37-CB aerogel, Raman spectra were used to analyze the O–H stretching vibration region with Gaussian fitting. As shown in Fig. 3l, the peaks at 3196 and 3301 cm^−1^ correspond to FW, representing water molecules with complete hydrogen bonding. The peaks at 3455 and 3606 cm^−1^ correspond to IW, representing water molecules with weak hydrogen bonding [63]. The experimental results showed that the IW/FW ratio was 0.73. This indicates that the CA37-CB aerogel contains a large amount of IW. Compared with FW, IW has weaker hydrogen bonding, lower surface tension, and a larger evaporation interface area. Thus, its evaporation enthalpy decreases significantly, and the corresponding evaporation rate increases rapidly [64]. This is because the hydrophilic groups in CMCS and SA strengthen the interactions with water, optimize the confinement environment within the porous network, and promote the transition of water into the intermediate state, thereby lowering the effective evaporation enthalpy [65].

To further verify the effect of the aerogel network on the evaporation enthalpy, DSC and dark evaporation experiments were conducted for CA37-CB. As shown in Fig. 3m, pure water exhibited a sharp endothermic peak in the DSC. The heat flow signal dropped rapidly at the peak. The CA37-CB aerogel showed a broad endothermic peak, indicating a change in the water distribution [66]. The calculated results show that pure water requires 2481 J g^−1^ for evaporation, whereas the CA37-CB aerogel requires only 968 J g^−1^.

This means the energy demand per unit mass of water is greatly reduced, which helps achieve photothermal evaporation rates higher than the theoretical limit [56]. It should be noted that DSC-derived enthalpy reflects the thermodynamic state of confined or bound water under quasi-static heating conditions. Under DSC conditions, water evaporates completely in a relatively closed testing environment, which is different from the continuous water supply and dynamic evaporation in actual solar evaporation [67, 68]. In contrast, the dark evaporation-derived value reflects the effective evaporation behavior under dynamic evaporation conditions. Therefore, to be closer to real solar evaporation, a dark evaporation experiment was carried out to estimate the equivalent evaporation enthalpy of water in the CA37-CB aerogel. As shown in Fig. 3n, the equivalent evaporation enthalpy of CA37-CB aerogel derived from dark evaporation is 1197 J g^−1^, which is much lower than the theoretical evaporation enthalpy used for 3.5 wt% NaCl solution under the corresponding experimental conditions (2393 J g^−1^) [69]. This further confirms the direct relationship between intermediate water activation in CA37-CB aerogel and its high evaporation performance.

### Salt Resistance and Stability

Salt accumulation is a key challenge in photothermal seawater desalination processes. This significantly affects the service life, energy consumption, and water production of evaporators [70]. Inspired by the seawater filtration of fish gills, the CA37-CB biomass aerogel evaporator introduces CMCS and SA rich in anionic groups into the molecular design. This not only promotes the enrichment of intermediate water and reduces evaporation enthalpy, but also provides strong ion repulsion ability and hydrophilicity. Consequently, the material exhibited high salt resistance and long-term stability.

**Fig. 4:**
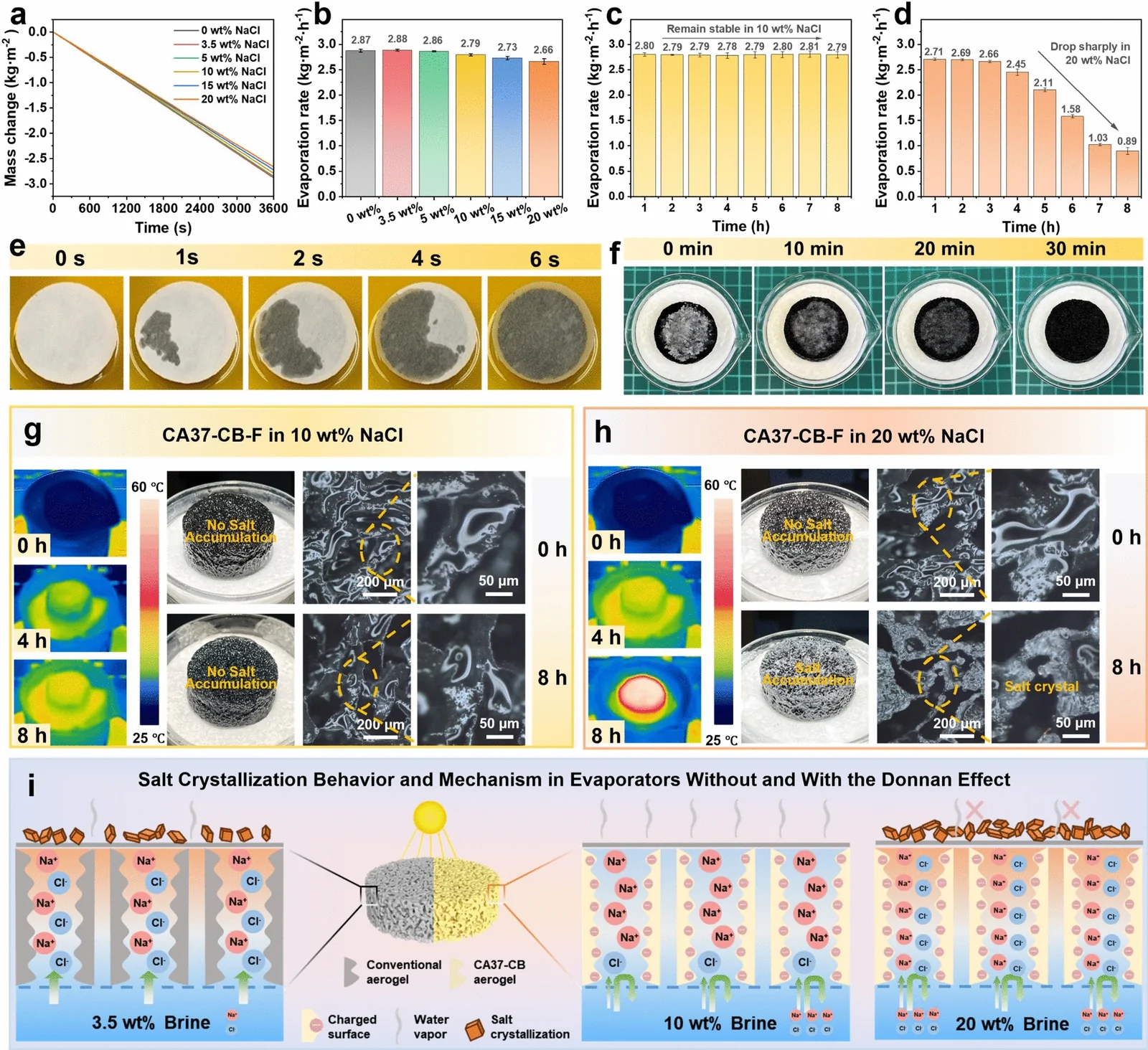
**a**, **b** Mass loss and evaporation rate of CA37-CB biomass aerogel evaporator in simulated seawater with different concentrations. **c** Continuous evaporation rate of CA37-CB-F evaporator in 10 wt% NaCl for 8h. **d** Continuous evaporation rate of CA37-CB-F evaporator in 20 wt% NaCl for 8h. **e** Water absorption ability of CA37-CB-F evaporator. **f** Dissolution of salt crystals on the surface of CA37-CB-F evaporator. Macroscopic, microscopic, and infrared thermal images of CA37-CB-F evaporator before and after continuous evaporation **g** in 10 wt% NaCl and **h** in 20 wt% NaCl. **i** Salt resistance mechanism of CA37-CB-F evaporator based on Donnan effect

Figure 4a, b shows the mass loss and evaporation rates of CA37-CB biomass aerogel evaporator at different salt concentrations. The evaporation rates in 0, 3.5, 5, 10, 15, and 20 wt% NaCl are 2.87, 2.88, 2.86, 2.79, 2.73, and 2.66 kg m^−2^ h^−1^. The results indicate that the evaporation rate of the 3.5% NaCl solution is slightly higher than that of pure water, which may be caused by the promotion effect of calcium ions in the aerogel [71]. With an increase in salt concentration, the evaporation rate of the CA37-CB aerogel evaporator remained relatively stable, with only a small decrease in the 20% NaCl solution. This is normal, because a higher salt concentration lowers the vapor pressure and hinders evaporation [70]. As shown in Fig. 4e, the structure during the simulated evaporation process was tested by placing the hydrophilic foam transport layer in a dish with methyl orange solution, and placing the CA37-CB aerogel on the foam. Dry white filter paper was placed on the surface of the CA37-CB aerogel. After only 6s, water was rapidly transported from the bottom to the top and wetted the filter paper, proving the fast water transport ability of the hydrophilic foam and CA37-CB aerogel. Moreover, a sufficient and continuous water supply is a key factor for enhancing the interfacial evaporation rate [72]. In addition, 1 g of solid NaCl was placed on top of the CA37-CB-F evaporator (Fig. 4f). After 20 min, the most of the NaCl solid on the surface dissolved, and after 30 min, the salt was completely dissolved. The rapid salt backflow phenomenon of the CA37-CB evaporator results from the combined effect of strong hydrophilicity and gravity [73].

Based on the excellent salt resistance of CA37-CB biomass aerogel, long-term stable operation tests were carried out. As shown in Fig. 4c, CA37-CB-F evaporator can continuously evaporate 10 wt% NaCl solution for 8h at a stable rate of about 2.79 kg m^−2^ h^−1^ without noticeable decline. Fig. 4g records the macroscopic, microscopic, and infrared thermal images of CA37-CB-F evaporator before and after continuous evaporation in 10 wt% NaCl. At the beginning of evaporation, the surface temperature of CA37-CB aerogel is close to 25 °C, and both the surface and the interior are in a wet state. With the progress of photothermal evaporation, the surface temperature rises to 40 °C after 4h and 42 °C after 8h. At this stage, the surface of CA37-CB aerogel is still wet, proving the feasibility of continuous evaporation in high-salinity brine.

To clarify the origin of this salt resistance, the surface charge characteristics of the aerogel network were evaluated by zeta potential measurements (Figs. S5 and S6). CA37 and CA37-CB exhibited strongly negative zeta potentials of − 56.12 and − 60.35 mV, respectively, confirming the abundant fixed anionic sites in the CMCS/SA network. In contrast, agar (AG), a neutral natural polysaccharide used as the non-Donnan control, showed a near-neutral zeta potential of 2.17 mV. This distinct difference demonstrates that the CMCS/SA-based CA37-CB aerogel possesses a negatively charged interfacial environment, while AG lacks effective fixed-charge-mediated ion regulation. This negatively charged network allows the salt resistance of CA37-CB-F to be explained by Donnan-type ion regulation (Fig. 4i).

For traditional evaporators without anionic groups, Na^+^ and Cl^–^ in seawater are transported upward with the water flow during long-term evaporation. When water evaporates, the local salt concentration becomes supersaturated, and salt crystallizes inside or on the surface of the absorber. This reduces the evaporation performance and blocks the water supply channels, leading a decline in performance and a shortened service life [70]. In this study, inspired by fish gills, the specially designed CA37-CB biomass aerogel contains many anionic groups, which strengthen the Donnan effect of the evaporator. Therefore, in 10 wt% NaCl solution containing Cl^–^ and Na^+^, the negatively charged CA37-CB biomass aerogel can limit the diffusion of Cl^–^ into the evaporator, while selectively attracting Na^+^. This lowers the local salt concentration below saturation and avoids salt crystallization [29, 74]. The selective adsorption or repulsion of ions enhances the salt resistance of the CA37-CB-F evaporator and supports its continuous evaporation in high-salinity brine.

However, ultra-high salinity still challenges the performance of CA37-CB-F evaporator. As shown in Fig. 4d, when CA37-CB-F evaporator was used for continuous evaporation of 20 wt% simulated seawater, it maintained a stable rate of 2.68 kg m^−2^ h^−1^ in the first 4h. The rate decreased sharply at 6h and almost lost photothermal evaporation ability after 8h. Figure 4h also records the macroscopic, microscopic, and infrared thermal images before and after continuous evaporation in 20 wt% NaCl. At the beginning, the surface and interior of CA37-CB aerogel were wet, similar to the 10 wt% NaCl case. But at 4h, the surface temperature reached 42 °C. This was because salt deposition on the surface reduced heat transfer efficiency and slowed heat transport inside the evaporator [75]. After 8h of evaporation, the surface temperature reached 60 °C, indicating that the aerogel had almost lost water. At this time, the microstructure of CA37-CB aerogel showed that the top surface was almost completely dry, with a large amount of salt crystallization. This result matches the evaporation rate change shown in Fig. 4d. Although the Donnan effect plays a key role in rejecting salt ions, it cannot handle brine with more than 15% salt concentration [17, 29]. In 20 wt% NaCl solution, excessive Cl^–^ and Na^+^ ions exist. The anionic groups of CA37-CB aerogel can only repel part of the Cl^–^. The remaining Cl^–^ diffuses together with Na^+^ to the top of the evaporator, where salt crystallizes and blocks continuous evaporation (Fig. 4i). Therefore, to achieve long-term treatment of very high-salinity water, other salt resistance strategies are needed to assist the Donnan effect.

**Fig. 5:**
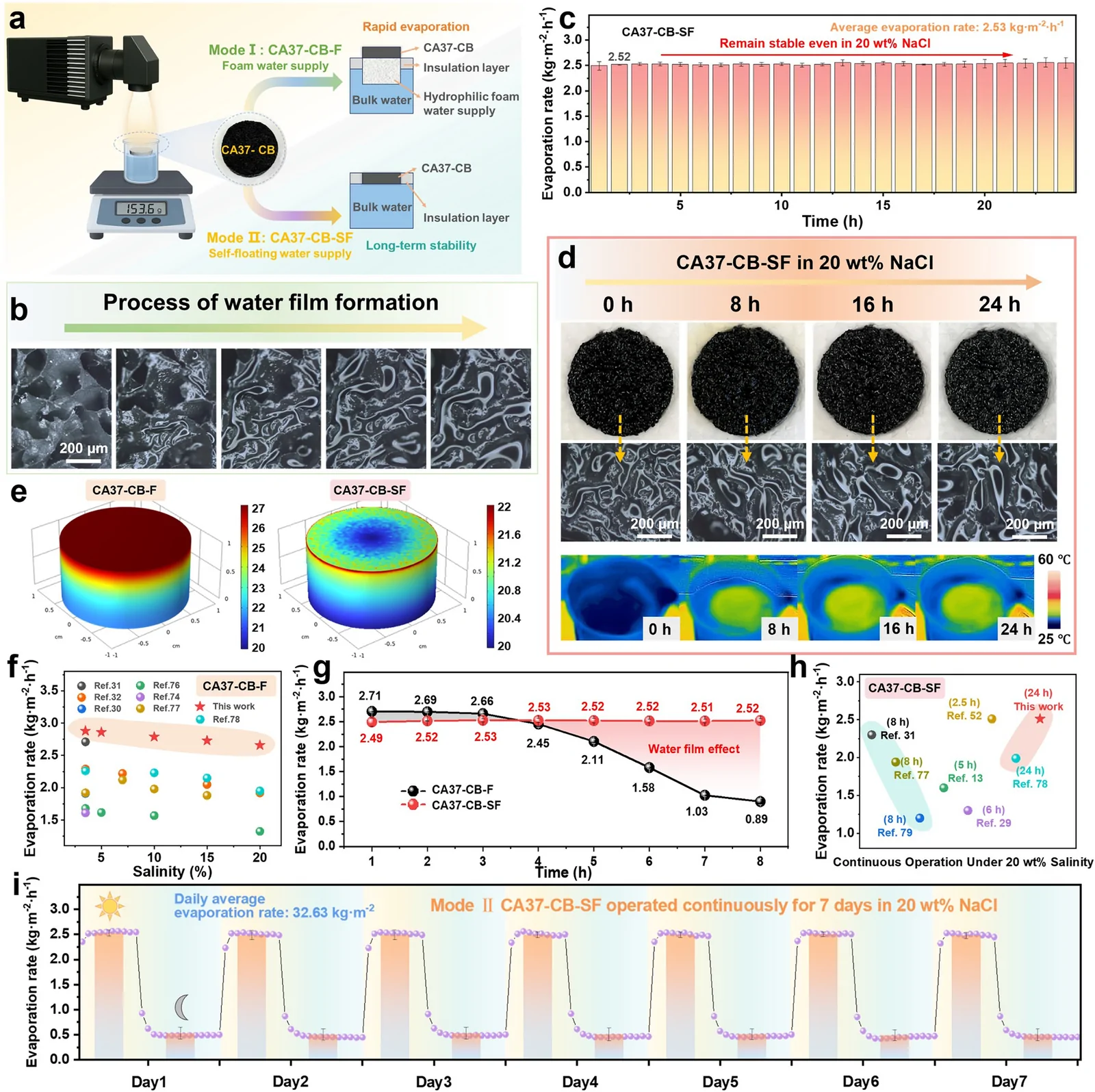
**a** Solar evaporation experimental setup of fish gill-inspired biomass aerogel evaporator with two operating modes. **b** Water film formation process of CA37-CB-SF evaporator. **c** CA37-CB-SF evaporator with dual-effect enhancement shows excellent 24 h continuous stable evaporation in 20 wt% NaCl. **d** Macroscopic, microscopic, and infrared thermal images of CA37-CB-SF evaporator during 24-h continuous evaporation in 20 wt% NaCl. **e** Simulated salt distribution of the single-effect CA37-CB-F evaporator and the dual-effect CA37-CB-SF evaporator. **f** Comparison of evaporation rates of the single-effect CA37-CB-F evaporator and several reported evaporators under different salinities. **g** Comparison of evaporation rate and long-term stability of the single-effect CA37-CB-F evaporator and the dual-effect CA37-CB-SF evaporator in 20 wt% NaCl. **h** Comparison of the dual-effect CA37-CB-SF evaporator with several reported evaporators in 20 wt% NaCl in terms of evaporation rate and long-term stability. **i** Seven-day continuous evaporation test of the dual-effect CA37-CB-SF evaporator in 20 wt% NaCl with 10h sunlight and 14h darkness per day

The anionic groups in the CA37-CB biomass aerogel network triggered the Donnan effect, relieved salt crystallization, and allowed continuous operation in 10 wt% high-salinity brine. However, the single-effect mode based on the Donnan effect still cannot handle 20 wt% ultra-high-salinity brine. To overcome this limitation, a fish gill-inspired dual-effect enhanced salt-free evaporation system named CA37-CB-SF was proposed. As shown in Fig. 5a, the evaporator based on the CA37-CB biomass aerogel has two modes. Mode I is CA37-CB-F (foam water supply, less water on the aerogel surface, and a higher evaporation rate). Mode II is CA37-CB-SF (aerogel directly floats on water, a liquid water film forms on the surface, and the evaporation rate is lower).

To further quantify the enhanced water replenishment in Mode II, the water transport rate and water mass transfer rate were measured (Fig. S7). The water transport rate of CA37-CB-SF reached 201% of that of CA37-CB-F, whereas its water mass transfer rate reached 244% of that of CA37-CB-F. These results indicate that Mode II provides faster and more continuous water replenishment, favoring the formation and renewal of the interfacial water film. This dynamic water film can locally dilute accumulated salts and assist the Donnan effect during high-salinity evaporation.

This enhanced water replenishment was also visually confirmed using the dye-transport test. As shown in Fig. S8, in the CA37-CB-SF mode, the CA37-CB biomass aerogel was directly placed in a dish containing the Rhodamine B solution. When a dry white filter paper was placed on its surface, water was transported from the bottom to the top within 4s. The transport speed was faster than that of CA37-CB-F in Mode I, proving the excellent hydrophilicity of the CA37-CB biomass aerogel. Similarly, when 1 g of NaCl solid was placed on top of the CA37-CB-SF evaporator (Fig. S9), the salt crystals completely dissolved in only 9 min. This shows a strong salt backflow ability. In Mode II, when the aerogel contacted bulk water, a flowing water film gradually formed on the surface (Fig. 5b), activating the water film effect, which continuously diluted the local salt concentration and assisted the Donnan effect.

Therefore, when the water film effect and the Donnan effect act together, the fish gill-inspired dual-effect CA37-CB biomass aerogel shows strong stability for evaporation in 20 wt% ultra-high-salinity brine. As shown in Fig. 5c, the CA37-CB-SF evaporator continuously operated in 20 wt% NaCl solution for 24 h, with a stable evaporation rate of 2.53 kg m^−2^ h^−1^ and no decline. In contrast to the CA37-CB-F evaporator, which shows salt crystallization after 8 h (Fig. 4h), the CA37-CB-SF evaporator maintained a wet water film state after 24 h of continuous operation, with no salt crystallization (Fig. 5d). Infrared thermal images indicate that the surface temperature remained at approximately 38 °C, demonstrating that the system retained sufficient water supply for long-term evaporation. Numerical simulations (Note S2 and Table S1) were performed for the salt distribution of the single-effect CA37-CB-F evaporator and dual-effect CA37-CB-SF evaporator in a 20 wt% NaCl solution for 5 h. As shown in Figs. 5e and S10, the steady-state salt concentration on the surface of the CA37-CB-F evaporator exceeded 26 wt%, causing salt precipitation. In contrast, the steady-state salt concentration on the surface of the CA37-CB-SF evaporator through the synergy between the Donnan effect and the water-film effect was maintained at 22 wt%, which allowing continuous evaporation without salt crystallization. The simulation results were consistent with the experimental data.

Figure 5g compares the evaporation performance of the CA37-CB-F evaporator in Mode I and the CA37-CB-SF evaporator in Mode II in 20 wt% ultra-high-salinity brine. During the first 3 h, the CA37-CB-F evaporator exhibited a higher evaporation rate than CA37-CB-SF, because the photothermal heating and water supply in Mode I were more balanced during short term evaporation. However, after 3 h, the CA37-CB-F evaporator began to accumulate salt crystals owing to the absence of a flowing water film. By 8 h, the evaporation rate dropped rapidly to 0.89 kg m^−2^ h^−1^. In contrast, the CA37-CB-SF evaporator maintained continuous water transport and efficient evaporation under the support of the water film effect, demonstrating excellent salinity tolerance and reliable long-term performance.

To further distinguish the contributions of the water-film effect and the Donnan effect, an AG-CB-SF evaporator, prepared by replacing the charged CMCS/SA network with near-neutral agar (AG), was used as a water-film-only control under the same Mode II configuration (Fig. S11). Although AG-CB-SF could initially form a surface water film, its evaporation rate in 20 wt% NaCl decreased sharply from 2.36 to 1.36 kg m^−2^ h^−1^ after only 3 h and further dropped to about 1.16 kg m^−2^ h^−1^ during prolonged operation. Macroscopic, microscopic, and infrared thermal images revealed that the initially hydrated surface gradually transformed into a salt-covered film, leading to blocked evaporation and increased surface temperature (Fig. S12). These results demonstrate that the water-film effect alone is insufficient to sustain salt-free evaporation under ultra-high salinity, and that Donnan-type ion exclusion is indispensable for the long-term stability of the dual-effect CA37-CB-SF evaporator.

This conclusion was further supported by quantitative salt accumulation analyses and microstructural characterizations. After 8 h of evaporation in 20 wt% NaCl, the top 2 mm of the dried evaporators, where salt accumulation was most severe, were collected for weighing. The calculated salt accumulation ratios of CA37-CB-F, CA37-CB-SF, and AG-CB-SF were 320%, 150%, and 592%, respectively (Fig. S13). Among them, the dual-effect CA37-CB-SF showed the lowest salt accumulation ratio, indicating that only a small amount of salt residue remained on the aerogel framework. FESEM and EDS mapping further revealed distinct salt deposition behaviors among the three evaporators (Fig. S14). In the single Donnan effect evaporator CA37-CB-F, thick salt blocks were formed on the aerogel walls. In contrast, the dual-effect CA37-CB-SF exhibited only a few dispersed residual deposits on the aerogel framework, with no obvious salt crystals, demonstrating salt-free evaporation. For the water-film-only control AG-CB-SF, a thick salt film directly covered the aerogel surface and blocked the porous channels of the aerogel. These results visually and quantitatively confirm the clear advantage of the fish gill-inspired dual-effect strategy in suppressing salt accumulation and maintaining an open evaporation interface under ultra-high salinity.

Therefore, the two operational modes of the fish gill-inspired CA37-CB aerogel can be selected according to practical needs. When treating medium to low salinity water below 10 wt%, Mode I (CA37-CB-F) is preferred because it enables rapid water production with an evaporation rate of 2.88 kg m^−2^ h^−1^. When treating highly concentrated brine around 20 wt%, Mode II (CA37-CB-SF) is more suitable because it sacrifices part of the evaporation rate but ensures long-term stable operation, including 24 h of uninterrupted desalination and 7 days of cycling. The ability to switch between these two modes significantly broadens the application scope of the fish gill-inspired biomass aerogel evaporator and provides practical adaptability for real desalination scenarios. Figure 5f compares the single-effect CA37-CB-F evaporator with several reported evaporators under different salinity conditions [30–32, 74, 76–78]. The CA37-CB-F evaporator delivers one of the highest evaporation rates among the reported systems, indicating that it is highly suitable for fast solar-driven evaporation under daily environmental conditions. Figure 5h compares the dual-effect CA37-CB-SF evaporator with representative evaporators reported for 20 wt% NaCl [13, 29, 31, 52, 77–79]. The CA37-CB-SF evaporator maintained stable evaporation for 24 h without noticeable decay, demonstrating competitive performance in the field of salt-resistant solar evaporation. Figure 5i further evaluates the long-term cycling stability of the CA37-CB-SF evaporator under practical conditions. The evaporator operated in a 20 wt% NaCl solution for 7 consecutive days under a daily cycle of 10h of sunlight and 14 h of darkness. Throughout the test, the evaporation rate remained stable at approximately 2.5 kg m^−2^ h^−1^ during sunlight and about 0.5 kg m^−2^ h^−1^ during the dark period. The average daily water production reached 32.63 kg m^−2^, indicating considerable application potential. These results confirm that the synergy of the Donnan effect and water film effect provides the CA37-CB-SF evaporator with robust long-term salt resistance under ultra-high-salinity conditions.

**Fig. 6:**
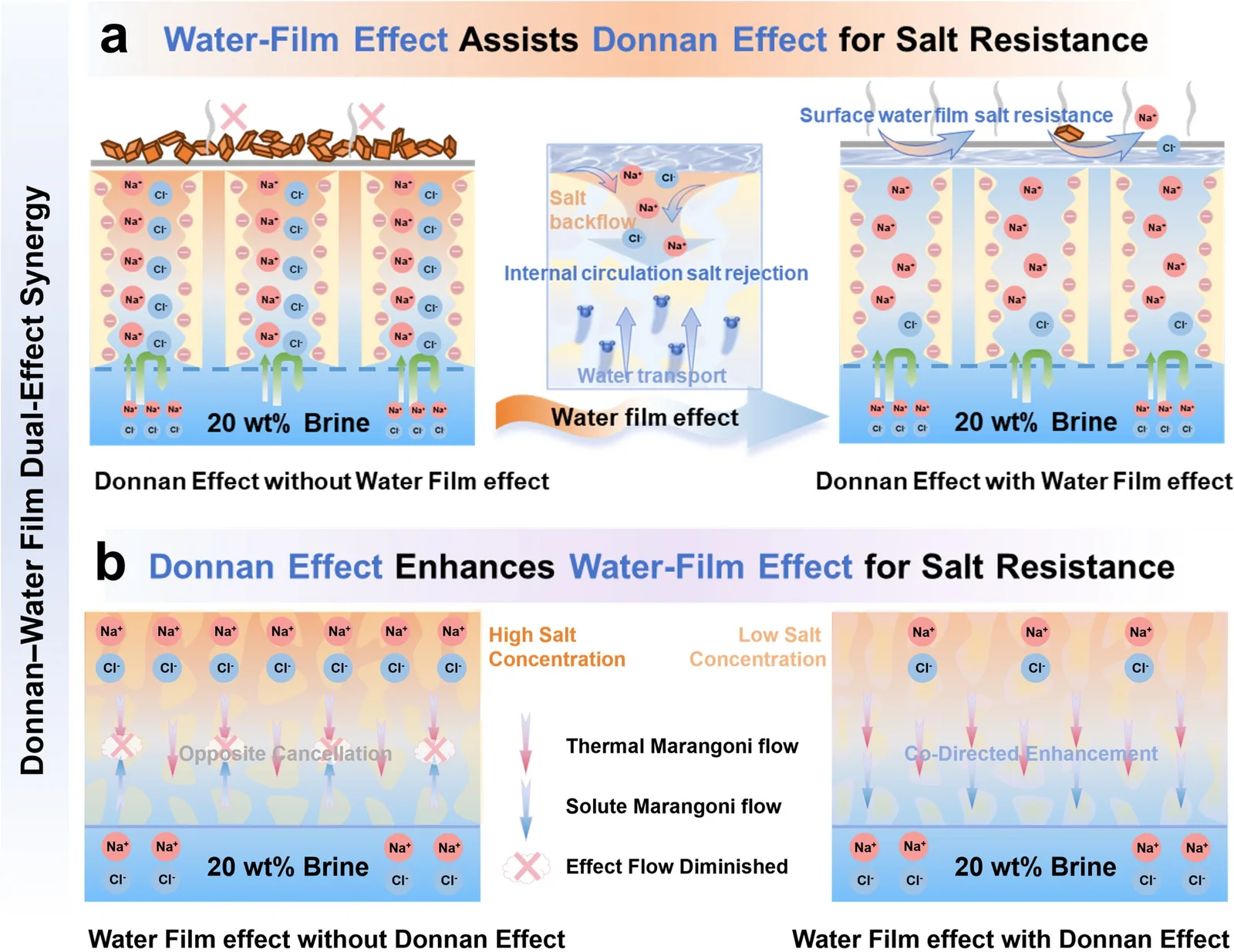
Salt resistance mechanism of CA37-CB-SF evaporator based on dual-effect synergy. **a** Water-film effect assists Donnan effect for salt resistance. **b** Donnan effect enhances water-film effect for salt resistance

In nature, marine fish can swim freely in seawater without accumulating excessive amounts of salt in their bodies. This ability primarily originates from the purification function of the gills (Fig. 1a). Through the combined action of ion-selective regulation and a dynamic water film, fish gills achieve efficient salt exclusion even in high-salinity environments. Inspired by this natural mechanism, the CMCS/SA biomass aerogel evaporator designed in this work exhibits a dual-effect salt resistance mechanism based on the synergy between the water film effect and the Donnan effect, as illustrated in Fig. 6.

First, the water film enhances the Donnan effect (Fig. 6a). Under photothermal excitation, a stable and continuously renewed water film is formed at the evaporation interface. This water film induces interfacial flow, ensures continuous water replenishment and dilutes the local ion concentration [67]. The flowing film also removes nascent salt crystals and prevents the formation of salt crusts. The maintained hydration keeps the functional groups active and prevents the surface from drying. Meanwhile, interfacial convection transports cations away from the surface, preserving the repulsive ability of the fixed negative groups. Therefore, the water film effect provides a stable operating environment that enables the Donnan effect to maintain long-term ion regulation.

Second, the Donnan effect reinforces the water film effect (Fig. 6b). The fixed negative groups reject anions and maintain a surface ion concentration that is much lower than that of the bulk solution. This creates a distribution pattern of low salinity at the interface and high salinity in the bulk, which is opposite to the typical pattern observed in conventional evaporators where interfacial salinity rapidly accumulates [80]. This interfacial salinity distribution was experimentally verified by monitoring the salinity of the surface water film and bulk brine during evaporation (Figs. S15 and S16). For the dual-effect CA37-CB-SF evaporator (Fig. S16a), the surface salinity remained consistently lower than that of the bulk brine, indicating that Donnan-type ion exclusion effectively suppressed interfacial salt enrichment [81, 82]. In contrast, for the water-film-only AG-CB-SF evaporator, the surface salinity rapidly exceeded the bulk salinity and increased sharply after 4 h, corresponding to the onset of salt crystallization (Fig. S16b). The reduced interfacial salinity suppresses salt nucleation and reverses the solute Marangoni flow, aligning it with the thermal Marangoni flow [31]. This significantly strengthens the interfacial water film convection and enhances the renewal of the evaporating surface.

Overall, the water film effect and the Donnan effect do not operate independently. They interact and mutually reinforce each other, forming a positive feedback loop. The Donnan effect lowers the interfacial salinity and creates favorable ionic gradients for water film formation. The water film effect continuously dilutes ions and maintains interfacial hydration, preserving the electrostatic potential required for effective Donnan regulation. This synergistic coupling produces a combined effect that is greater than the sum of individual contributions. Therefore, the fish gill-inspired biomass evaporator developed in this work achieves dynamic salt regulation, sustained water transport, and long-term stable operation under ultra-high-salinity conditions.

### Eco-Safe Solar Water Purification and Outdoor Experiments

**Fig. 7:**
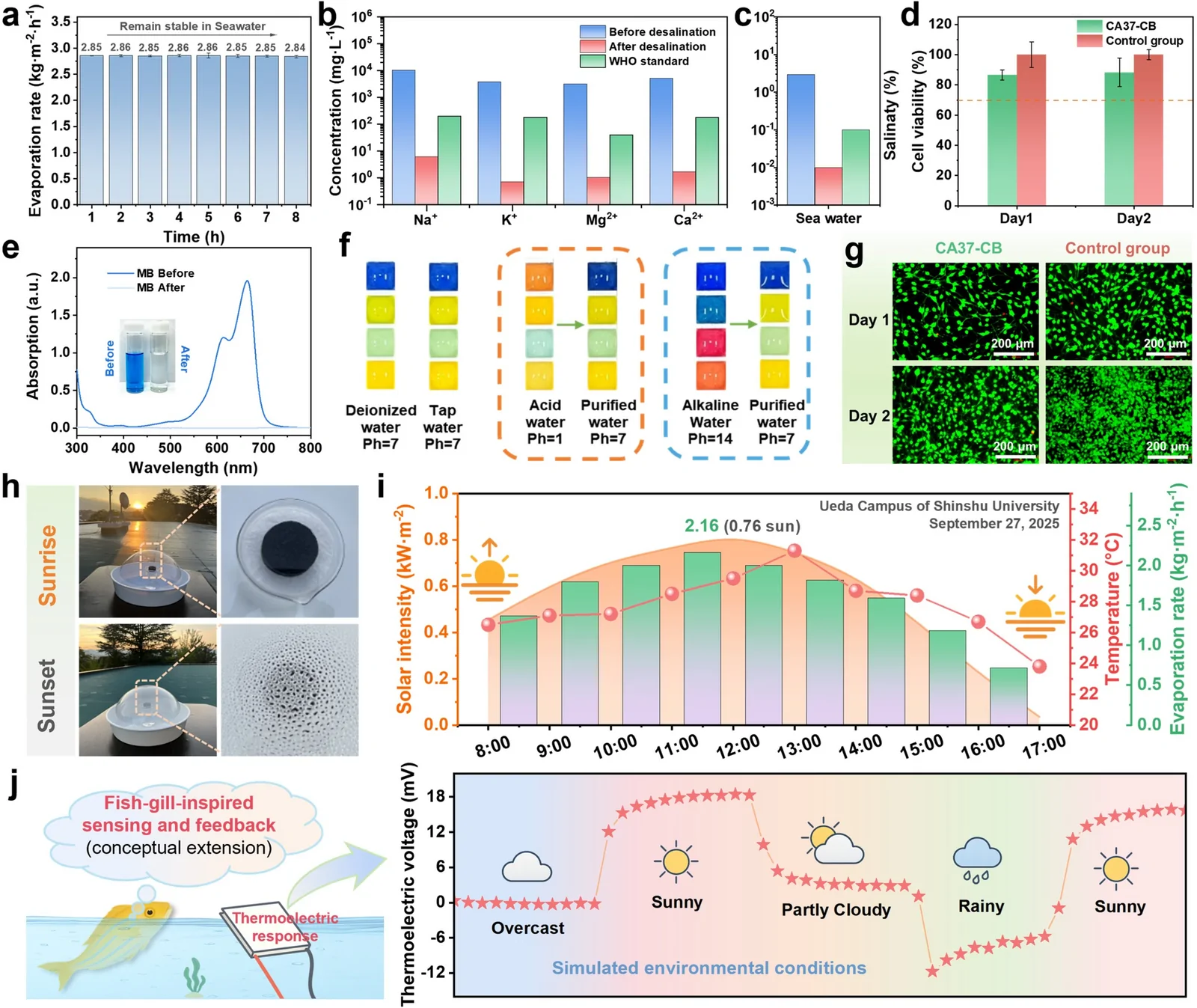
**a** Stable evaporation performance of the fish gill-inspired CA37-CB biomass aerogel evaporator in real seawater. **b**, **c** Changes in major metal ion concentrations and salinity of seawater before and after photothermal evaporation. Cytotoxicity evaluation of the CA37-CB aerogel evaporator: **d** cell viability of NIH 3T3 cells and **g** live/dead fluorescence images. **e** Removal capability of the CA37-CB aerogel evaporator for organic dyes. **f** Removal capability of the CA37-CB aerogel evaporator for acidic and alkaline wastewater. **h** Photograph of the outdoor evaporation setup from sunrise to sunset on the Shinshu University campus. **i** Real-world evaporation performance of the CA37-CB aerogel evaporator in outdoor conditions using natural seawater. **j** Conceptual extension of fish gill-inspired sensing for thermoelectric monitoring of environmental weather changes

The simulated seawater experiments above demonstrate the excellent salt resistance of the fish gill-inspired CA37-CB aerogel evaporator. Under daily conditions where rapid freshwater production is preferred, the evaporator is used in the CA37-CB-F mode. When applied to natural seawater, the evaporator also delivers strong photothermal evaporation performance. As shown in Fig. 7a, natural seawater collected from Yurihonjo, Akita, Japan (39°23′39.2″N, 140°00′40.4″E) was tested under 8 h of continuous illumination. The CA37-CB aerogel evaporator maintained a high evaporation rate of 2.85 kg m^−2^ h^−1^, confirming its effectiveness in practical seawater desalination. To further evaluate purification capability, the composition of seawater before and after evaporation was analyzed by inductively coupled plasma (ICP). The concentrations of the major ions (Na^+^, K^+^, Mg^2+^, and Ca^2+^) in the condensed water were far below the WHO drinking water limits [83, 84] (Fig. 7b). Salinity measurements using a salinometer show that the total salt content of seawater decreased by three orders of magnitude after purification (Fig. 7c). Electrical resistance measurements also support this finding. The resistances of natural seawater, purified water, tap water, and deionized water were 0.044, 0.178, 0.090, and 3.314 MΩ, respectively, indicating that the purified water approached the quality of tap water or DI water [85] (Fig. S17). The CA37-CB aerogel evaporator is also effective for wastewater remediation. Wastewater containing methylene blue (MB), methyl orange (MO), or rhodamine B (RhB) exhibited strong coloration before treatment, but became nearly colorless after evaporation. UV–vis spectra show that the absorbance values of the purified water dropped to almost zero (Figs. 7e and S18). Acidic wastewater (pH 1) and alkaline wastewater (pH 14) were also purified into neutral water during evaporation. The pH of the collected water approached 7 and was comparable to that of deionized and tap water (Fig. 7f). These results demonstrate that the CA37-CB aerogel evaporator has strong purification capacity and practical potential for diverse types of wastewaters [36].

As a biomass-based material, the CA37-CB aerogel evaporator also exhibits biosafety and biodegradability, which are important for clean water production and environmental protection. As shown in Fig. 7d and Note S3, the cytotoxicity was evaluated using NIH 3T3 fibroblasts. After 2 days of incubation, the CCK-8 assay showed a cell viability of 88%, which is higher than the commonly accepted safety threshold of 70% and indicates low cytotoxicity of the aerogel [86]. Live/dead fluorescence staining was further used to visualize cell viability (Fig. 7g). The NIH 3T3 cells showed predominantly green fluorescence, consistent with the CCK-8 results and confirming good cell viability, which further demonstrated the biosafety of the CA37-CB aerogel during use. The germination and growth of plant seeds are a widely used method for evaluating the toxicity of water [87]. Wheat seeds were cultivated using purified seawater obtained from the CA37-CB evaporator (Fig. S19). Due to the high salinity of seawater, no seeds germinated under direct irrigation with seawater. In contrast, seeds irrigated with purified water or tap water germinated on the 2nd day and showed rapid growth on the 3rd day. These results indicate that the purified water is non-toxic and suitable for potential drinking and agricultural applications. From a life-cycle perspective, the environmental impact of evaporators after disposal is also important. To assess this, CA37-CB aerogel samples were buried in natural soil for biodegradation testing (Fig. S20). Because CMCS and SA are biodegradable polysaccharides, the CA37-CB aerogel gradually decomposed in soil and almost completely disappeared after 60 days. These results demonstrate that the fish gill-inspired CA37-CB aerogel evaporator is safe during operation and environmentally benign after use. It can provide clean water without leaving persistent plastic residues, making it a sustainable and eco-friendly candidate for solar-driven desalination and wastewater purification applications.

The CA37-CB aerogel evaporator also demonstrates strong applicability for real outdoor operation. Outdoor evaporation tests were conducted on the Shinshu University campus in Ueda, Nagano, Japan, on September 27, 2025, during a clear autumn day (Fig. 7h). Throughout the experiment, the ambient temperature, solar intensity, and system status were continuously recorded. As shown in Fig. 7i, when the sun rose at 8:00 a.m., the ambient temperature was 26.5 °C and the solar intensity was only 0.45 sun. As the temperature and sunlight increased, the evaporation rate of the CA37-CB evaporator gradually rose and reached 2.16 kg m^−2^ h^−1^ at noon. Although autumn sunlight was relatively weak with a maximum intensity of only 0.76 sun, the evaporator still maintained a high evaporation rate, highlighting its strong outdoor adaptability. Even under low-light conditions, the CA37-CB aerogel shows efficient photothermal conversion and stable water production. To further evaluate outdoor adaptability, 4-day outdoor evaporation tests were conducted under different weather conditions for both Mode I (CA37-CB-F in seawater) and Mode II (CA37-CB-SF in 20 wt% NaCl) (Fig. S21). Both operating modes showed stable evaporation responses following the natural fluctuations of solar intensity and weather, further confirming the outdoor environmental adaptability and operational stability of the CA37-CB evaporator. In addition, after soaking in natural seawater under outdoor conditions for 20 days, the CA37-CB aerogel still maintained good compression recovery, wet state mechanical stability, and a high evaporation rate, demonstrating its structural durability and long-term usability in seawater (Figs. S22 and S23).

These results indicate that the CA37-CB evaporator is a promising candidate for solar-driven clean water production. In addition to ion regulation, natural fish gills possess environmental sensing capabilities, such as the ability to detect temperature changes and other external stimuli. Functional-layered and self-powered sensing systems have recently shown promising potential for stable and intelligent monitoring applications [88, 89]. Inspired by this multifunctionality, a thermoelectric module was integrated to conceptually demonstrate environmental sensing. As shown in Fig. 7j, the output voltage exhibited distinct step-like variations under different simulated weather conditions (overcast, sunny, partly cloudy, rainy, and sunny again), conceptually illustrating gill-like environmental perception. Furthermore, although thermoelectric outputs are typically small, thermoelectric modules have been widely explored in solar-driven evaporation systems [90], and their combination with gill-inspired sensing offers the potential for more intelligent environmental monitoring during operation.

## Conclusion

Inspired by the cooperative salt excretion of fish gills, a CMCS/SA all biomass aerogel evaporator was developed through the MISC strategy. The negatively charged interfacial layer and hydrophilic polymer chains work together to trigger Donnan exclusion and water-film driven Marangoni flow under solar heating, thus establishing a stable and efficient dual-effect mechanism for salt rejection. This study further reveals that neither Donnan exclusion nor the water-film effect alone is sufficient to sustain effective salt rejection and stable evaporation under extreme salinity, whereas their synergistic coupling is essential for achieving long-term and high-efficiency salt resistance. Benefiting from this cooperative mechanism, the evaporator delivered an evaporation rate of 2.88 kg m^−2^ h^−1^ under one sun and operated for 24 h in 20 wt% brine without salt deposition, demonstrating excellent salt resistance and long-term stability. The interpenetrated network of rigid CMCS and flexible SA provided strong mechanical performance and ensured structural stability in complex environments. In addition, the evaporator was fabricated entirely from biomass materials, enabling scalable, green, and biodegradable production methods. Overall, this work demonstrates the successful application of a fish gill-inspired dual-effect mechanism in solar evaporation and opens a new paradigm for sustainable freshwater production and water treatment under extreme salinity.

## Supplementary Information

Below is the link to the electronic supplementary material.

Supplementary file1 (DOCX 6495 KB)
