# Supplementary material for: Gill-Inspired Dual-Effect All-Biomass Aerogel Evaporator Overcoming Salt Accumulation for Sustainable Solar Desalination
Source: Nanomicro Lett. 2026 Jul 13;18:435. doi: 10.1007/s40820-026-02284-8 (PMC13365294; doi:10.1007/s40820-026-02284-8)
Supplement: Supplementary file 1 — Supplementary file1 (DOCX 6495 KB) [file 40820_2026_2284_MOESM1_ESM.docx]

Supporting Information for

**Gill-Inspired Dual-Effect All-Biomass Aerogel Evaporator Overcoming Salt Accumulation for** **Sustainable Solar Desalination**

Nuo Liu^1,2^, Chunqing Niu^1,2^, Xin Zhang^1,2^, Deqian Yang^3^, Weiyang Gu^3^, Guangyu Zhang^4^, Haifeng Zhang^4^*, Chunhong Zhu^1,2,5^*, Jian Shi^1,2,5^*

^1^ Graduate School of Medicine, Science and Technology, Shinshu University, Tokida 3-15-1, Ueda, Nagano 386-8567, Japan

^2^ Institute for Fiber Engineering and Science (IFES), Shinshu University, Tokida 3-15-1, Ueda, Nagano 386-8567, Japan

^3^ Graduate School of Science and Technology, Shinshu University, Tokida 3-15-1, Ueda, Nagano 386-8567, Japan

^4^ College of Textile and Clothing, Nantong University, Nantong, Jiangsu 226019, P. R. China

^5^ Faculty of Textile Science and Technology, Shinshu University, Tokida 3-15-1, Ueda, Nagano 386-8567, Japan

*Corresponding authors. E-mails: [shi@shinshu-u.ac.jp](file:///E:\Nano-Micro%20Letters\2026\文章编校21761%2021783-YJP\文章编校21761%2021783-YJP\NML-2026-21783-YJP\shi@shinshu-u.ac.jp) (Jian Shi); [zhu@shinshu-u.ac.jp](file:///E:\Nano-Micro%20Letters\2026\文章编校21761%2021783-YJP\文章编校21761%2021783-YJP\NML-2026-21783-YJP\zhu@shinshu-u.ac.jp) (Chunhong Zhu); [zhanghf@ntu.edu.cn](file:///E:\Nano-Micro%20Letters\2026\文章编校21761%2021783-YJP\文章编校21761%2021783-YJP\NML-2026-21783-YJP\zhanghf@ntu.edu.cn) (Haifeng Zhang)

**Supplementary Figures and Tables**


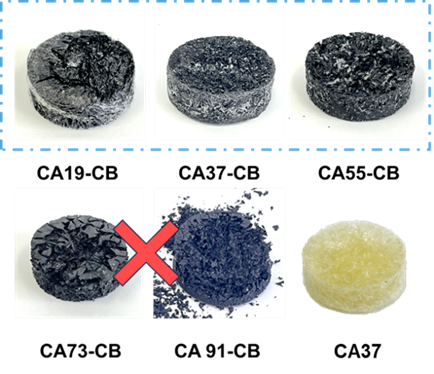


**Fig. S1** Macroscopic images of aerogel samples with different ratios.


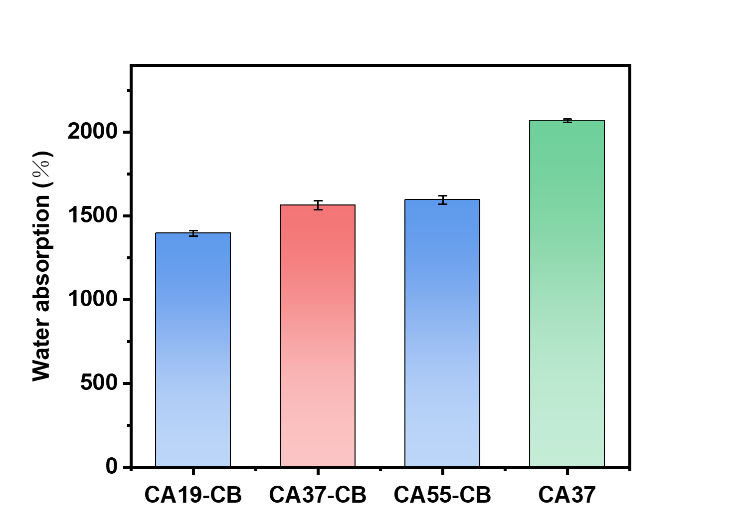


**Fig. S2** Water absorption performance of aerogel samples.


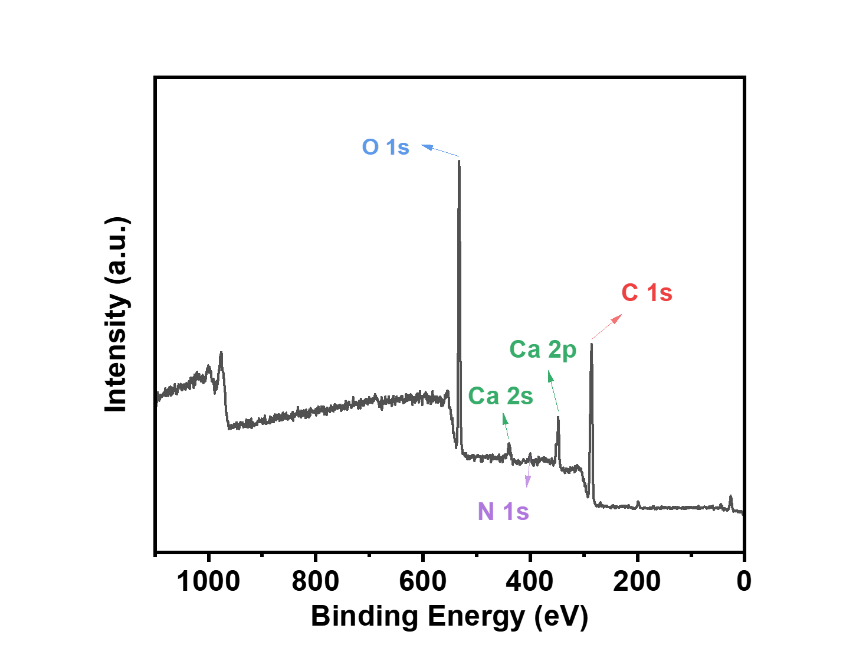


**Fig. S3** XPS analysis of the CA37-CB aerogel.


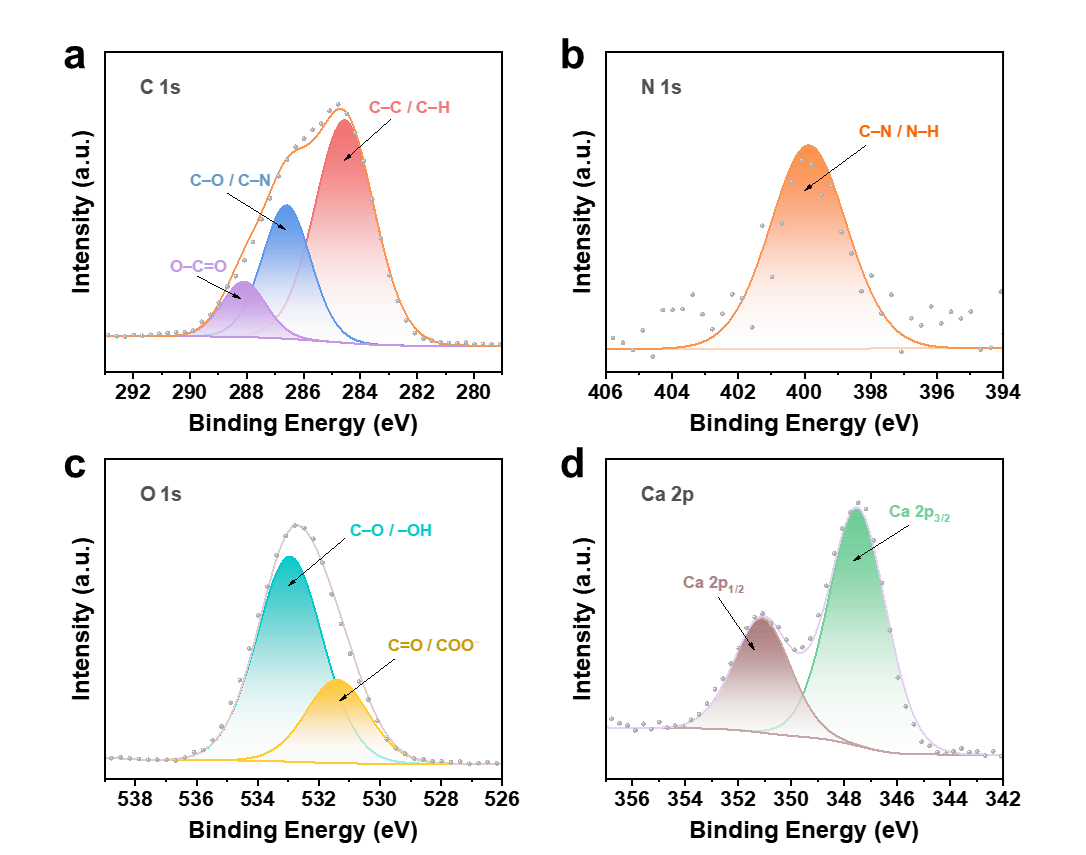


**Fig. S4** High-resolution XPS spectra of CA37-CB aerogel: C 1s (**a**), N 1s (**b**), O 1s (**c**), and Ca 2p (**d**).

**Fig. S5** Zeta potential values of CA37, CA37-CB, and AG.


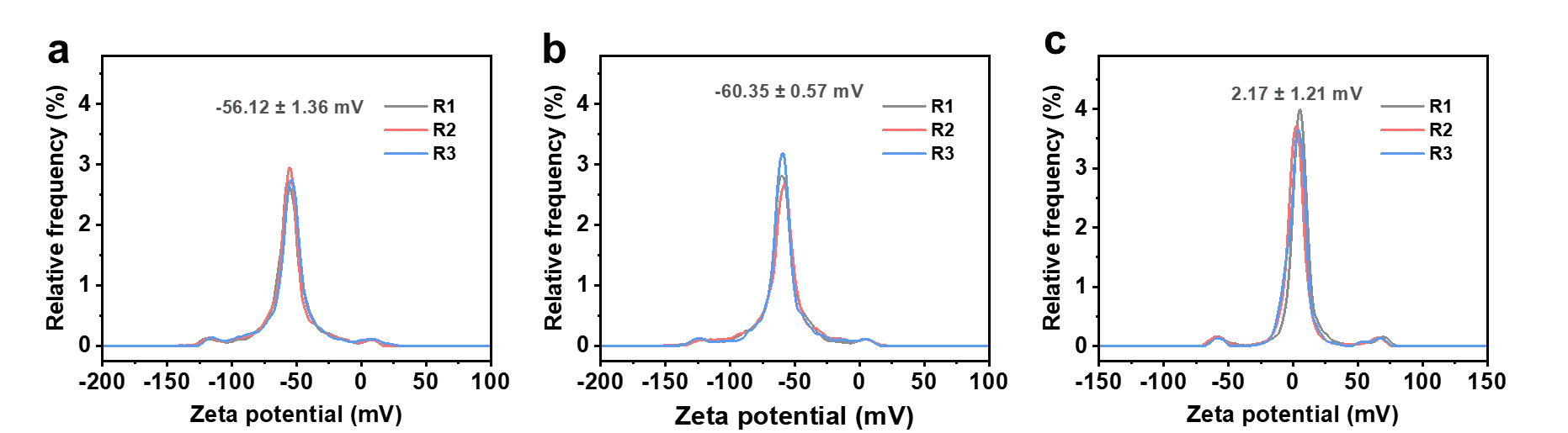


**Fig. S6** Representative zeta potential distribution curves of CA37 (**a**), CA37-CB (**b**), and AG (**c**).


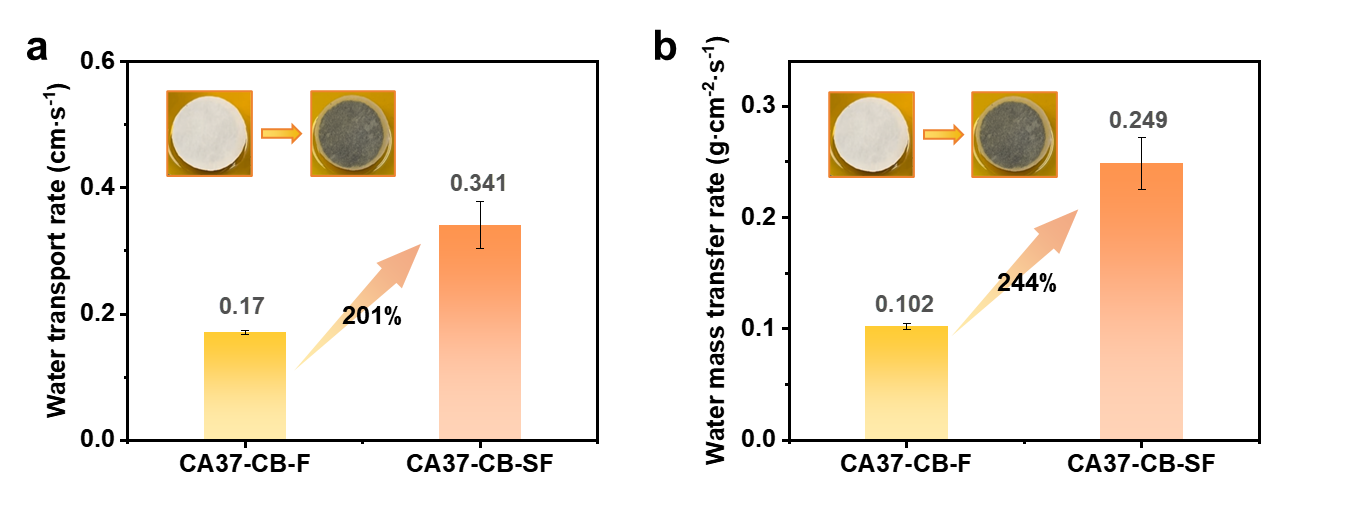


**Fig. S7** Water transport rate (**a**) and water mass transfer rate (**b**) of CA37-CB-F mode and CA37-CB-SF mode.


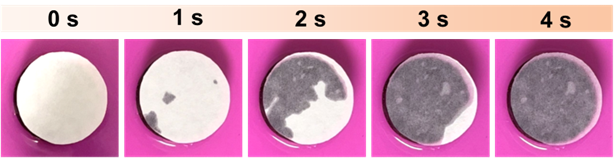


**Fig. S8** Water transport ability of the CA37-CB-SF evaporator.


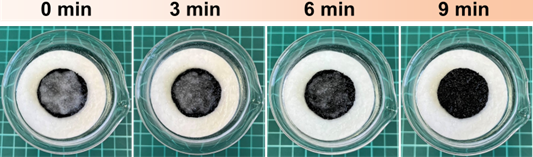


**Fig. S9** Dissolution of salt crystals on the surface of the CA37-CB-SF evaporator.


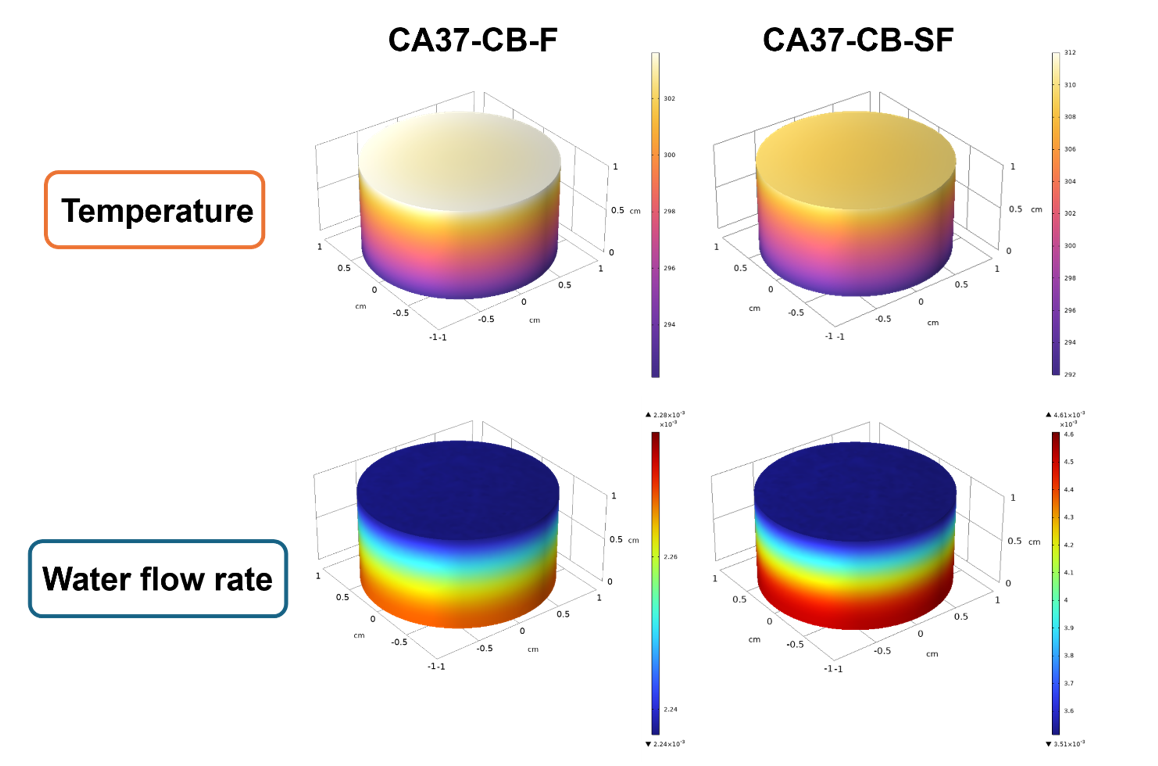


**Fig. S10** Numerical simulation of temperature and flow rate in CA37-CB-F and CA37-CB-SF evaporators.

**Fig. S11** Comparison of evaporation rate and long-term stability of the water-film-only AG-CB-SF evaporator and the dual-effect CA37-CB-SF evaporator in 20 wt% NaCl.


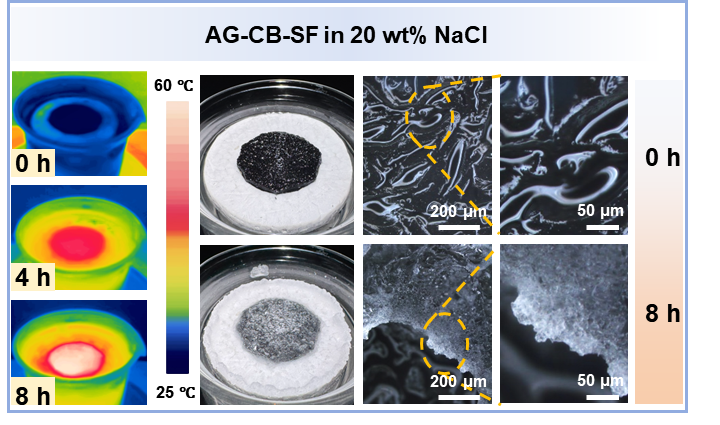


**Fig. S12** Macroscopic, microscopic and infrared thermal images of AG-CB-SF evaporator

before and after continuous evaporation in 20 wt% NaCl.


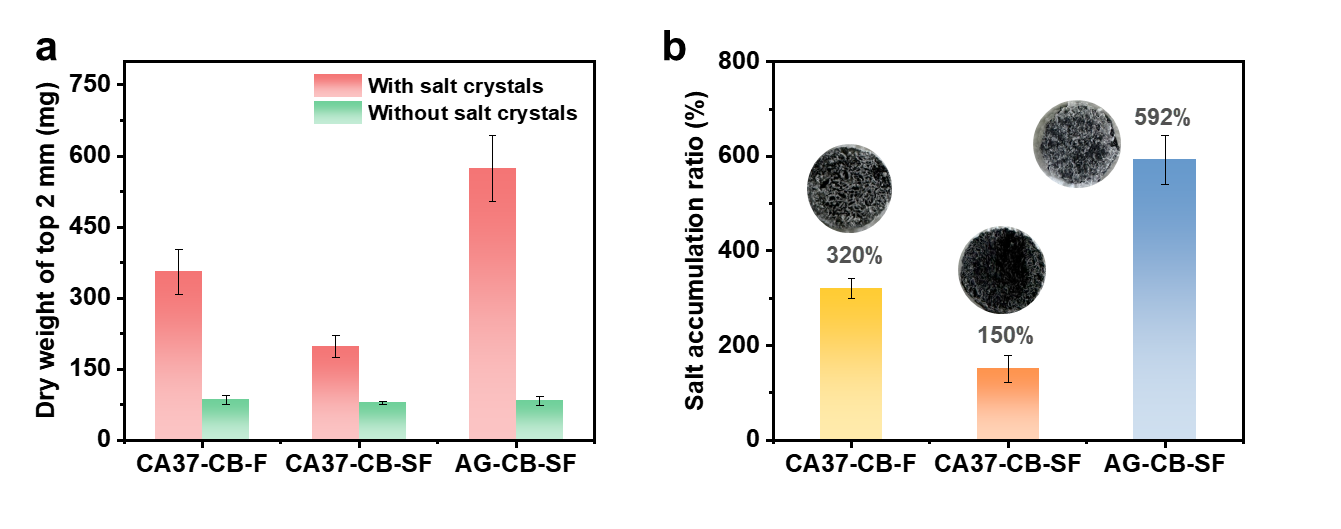


**Fig. S13** Quantitative comparison of surface salt accumulation on different evaporators after 8 h of evaporation in 20 wt% NaCl. (**a**) Dry weight of the top 2 mm of the evaporators before and after salt removal. (**b**) Calculated salt accumulation ratios of CA37-CB-F, CA37-CB-SF and AG-CB-SF.


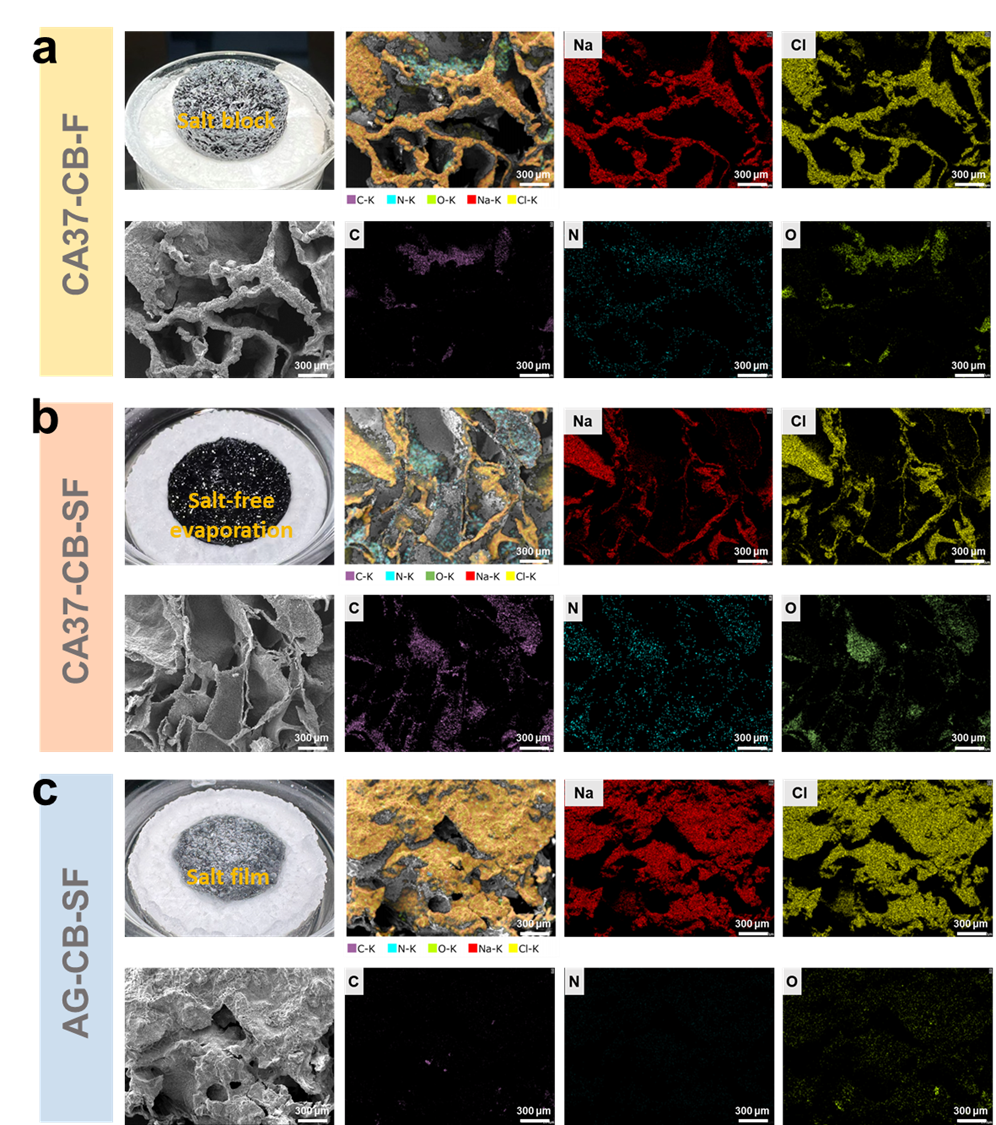


**Fig. S14** FESEM images and EDS elemental mapping of salt deposition on different evaporators after evaporation in 20 wt% NaCl: (**a**) CA37-CB-F, (**b**) CA37-CB-SF, and (**c**) AG-CB-SF.

**Fig. S15** Conductivity–salinity calibration curve used for salinity calculation.

Before conductivity measurement, the collected high-salinity surface and bulk brine samples were diluted by a fixed factor to ensure that the measured conductivity fell within the linear calibration range. The original salinity was then calculated by multiplying by the dilution factor.


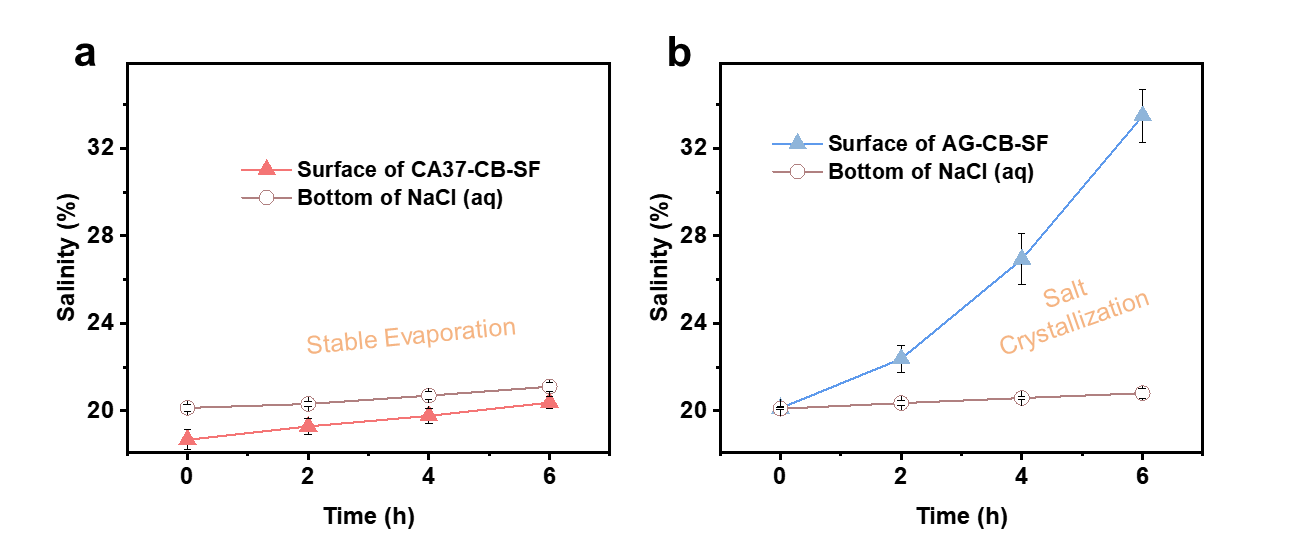


**Fig. S16** Time-dependent salinity changes in the surface water film and bulk brine of the dual-effect CA37-CB-SF evaporator (**a**) and the water-film-only AG-CB-SF evaporator (**b**) during evaporation in 20 wt% NaCl.


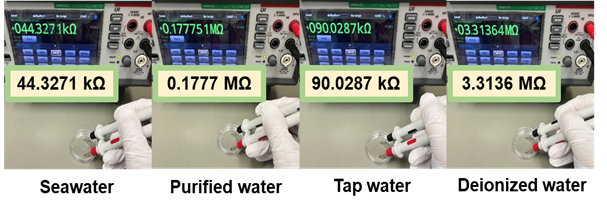


**Fig. S17** Electrical resistance of seawater before and after photothermal evaporation.


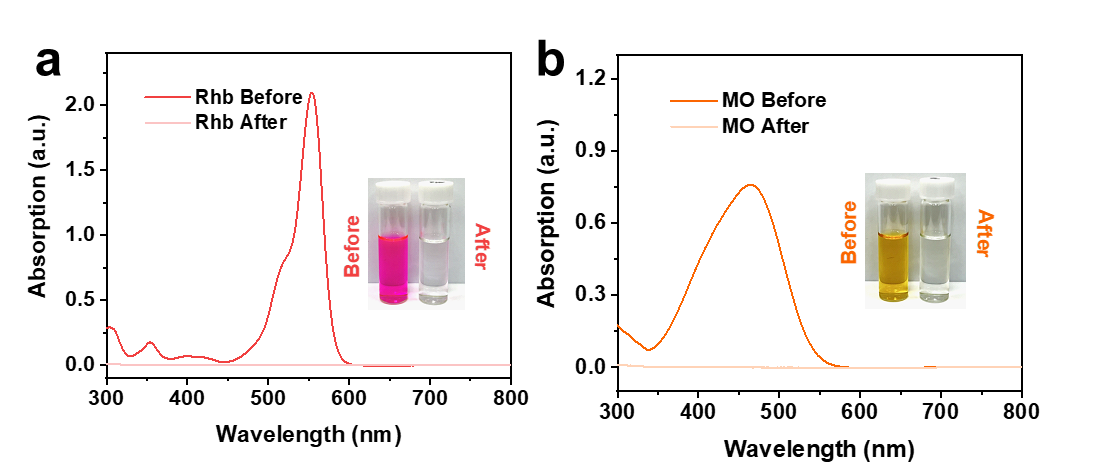


**Fig. S18** Removal ability of CA37-CB aerogel evaporator for organic dyes.


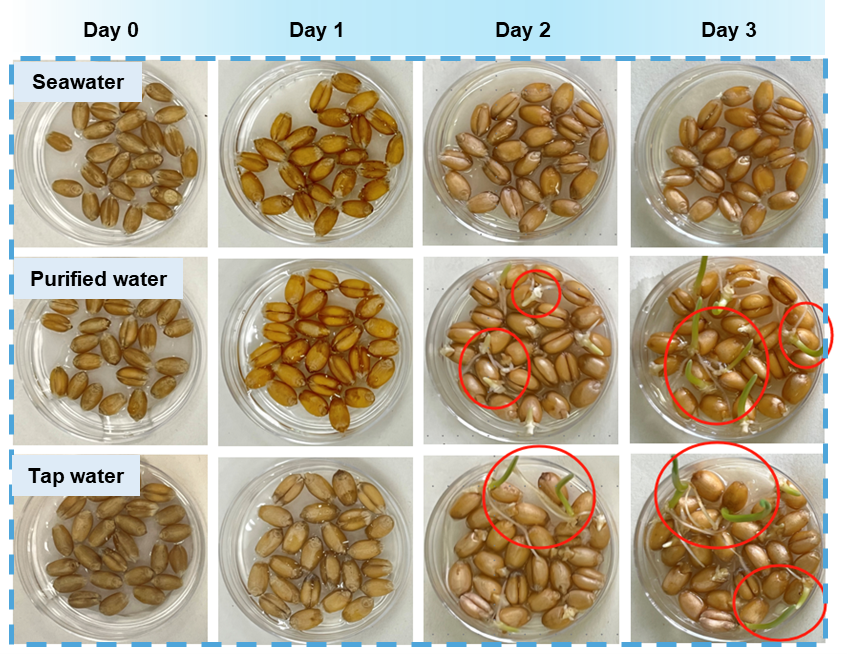


**Fig. S19** Germination of wheat seeds using purified water from the CA37-CB aerogel evaporator.


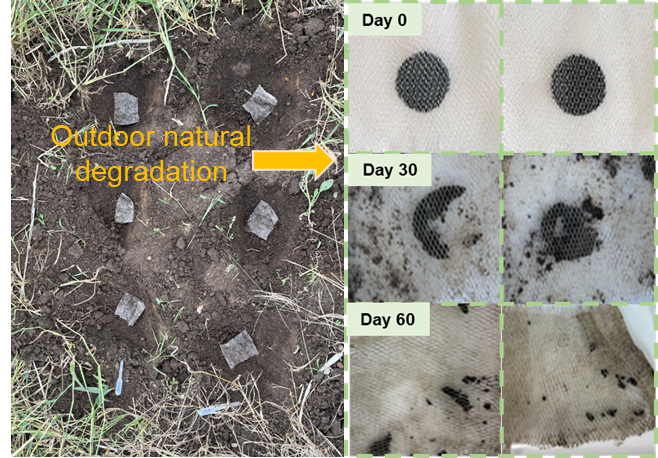


**Fig. S20** Soil degradation feasibility of the CA37-CB biomass aerogel evaporator.

**
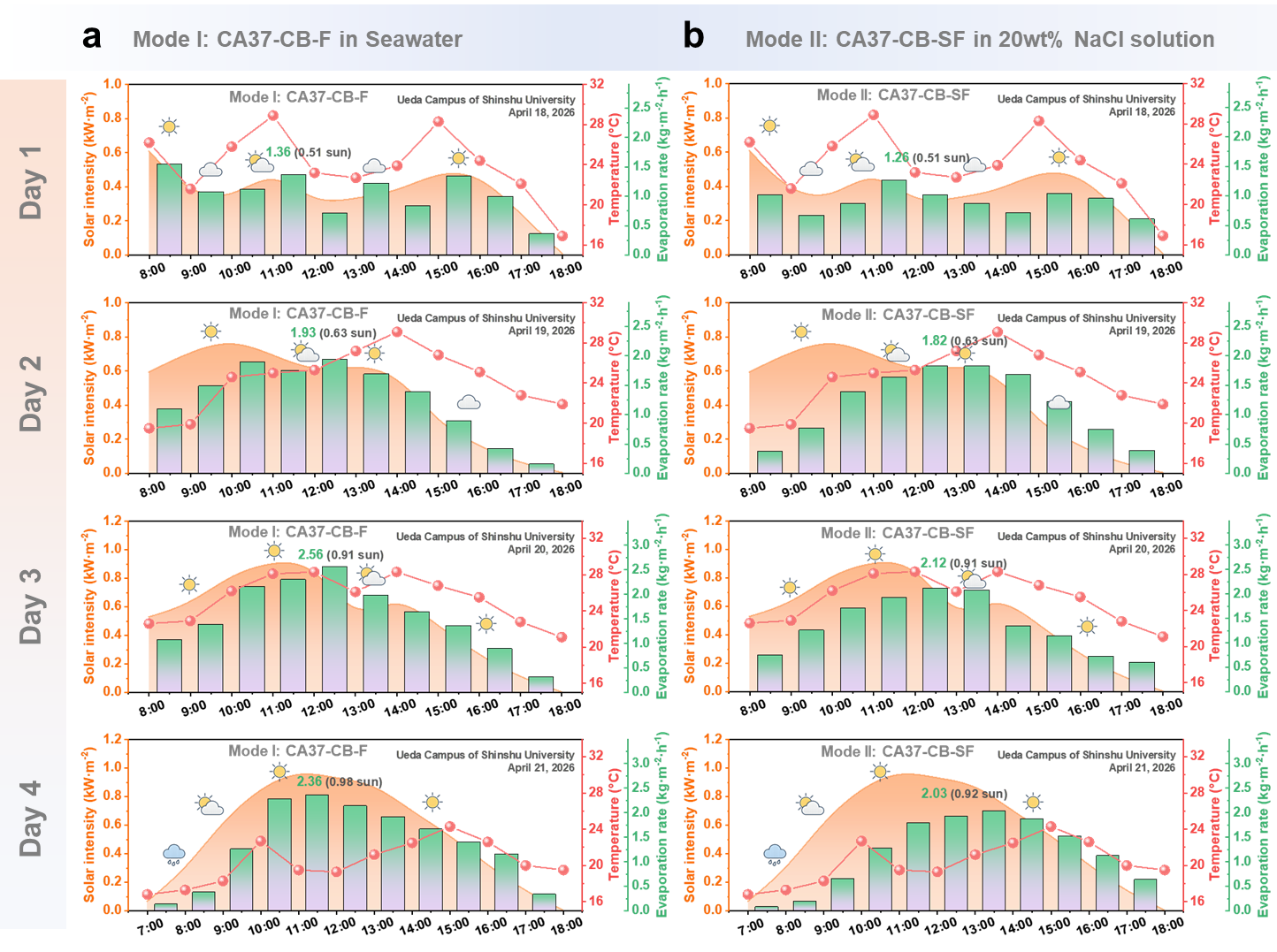
**

**Fig. S21** Outdoor environmental adaptability and stability of the CA37-CB evaporator over four consecutive days under different weather conditions. (**a**) Mode I: CA37-CB-F in seawater. (**b**) Mode II: CA37-CB-SF in 20 wt% NaCl solution.

During the four-day test, the weather conditions varied, including sunny, cloudy and rainy periods, resulting in natural fluctuations in solar intensity, ambient temperature and evaporation rate. In general, the evaporation rate increased with solar intensity and reached the highest value around noon. Both operating modes maintained stable evaporation behavior under outdoor conditions. On Day 3, when the solar intensity reached 0.91 sun at noon, Mode I achieved an evaporation rate of 2.56 kg m^-2^ h^-1^ in seawater, while Mode II achieved 2.12 kg m^-2^ h^-1^ in 20 wt% NaCl solution. These values are comparable to those obtained under laboratory conditions, demonstrating the strong outdoor adaptability and stability of the CA37-CB evaporator under different practical weather conditions.

**
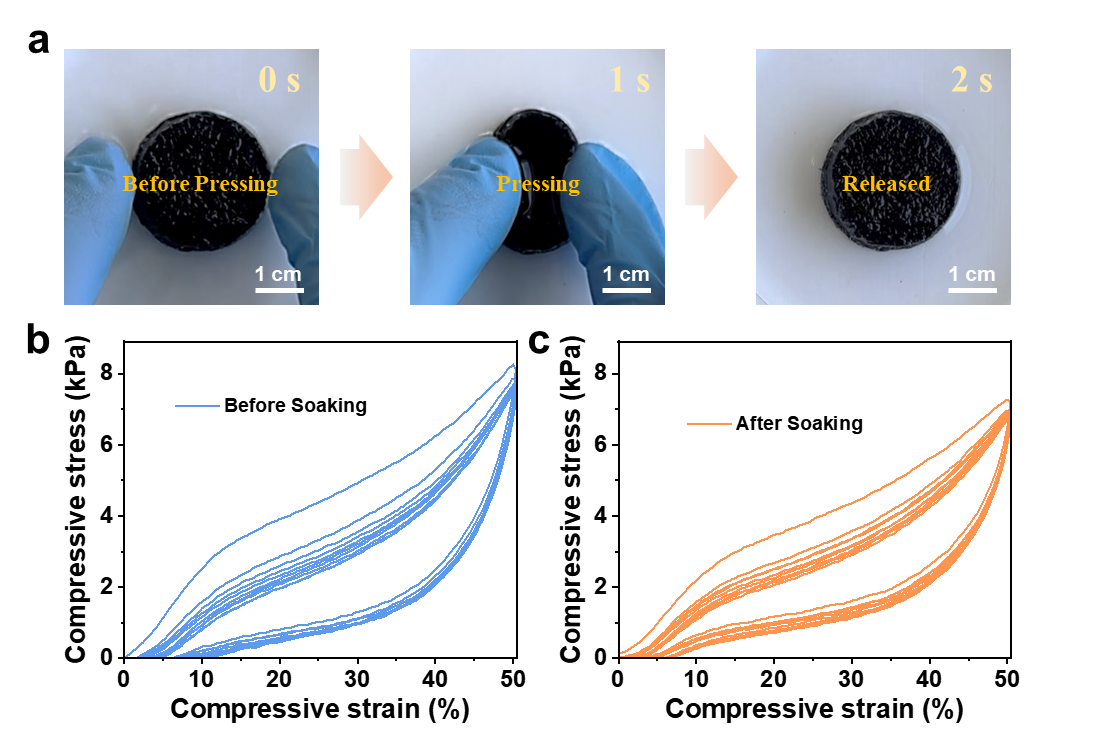
**

**Fig. S22** Compression recovery behavior of the CA37-CB aerogel after soaking in natural seawater under outdoor conditions for 20 days. Macroscopic recovery during transverse pressing and release (**a**), axial cyclic compression curves before soaking (**b**) and after soaking (c).

**
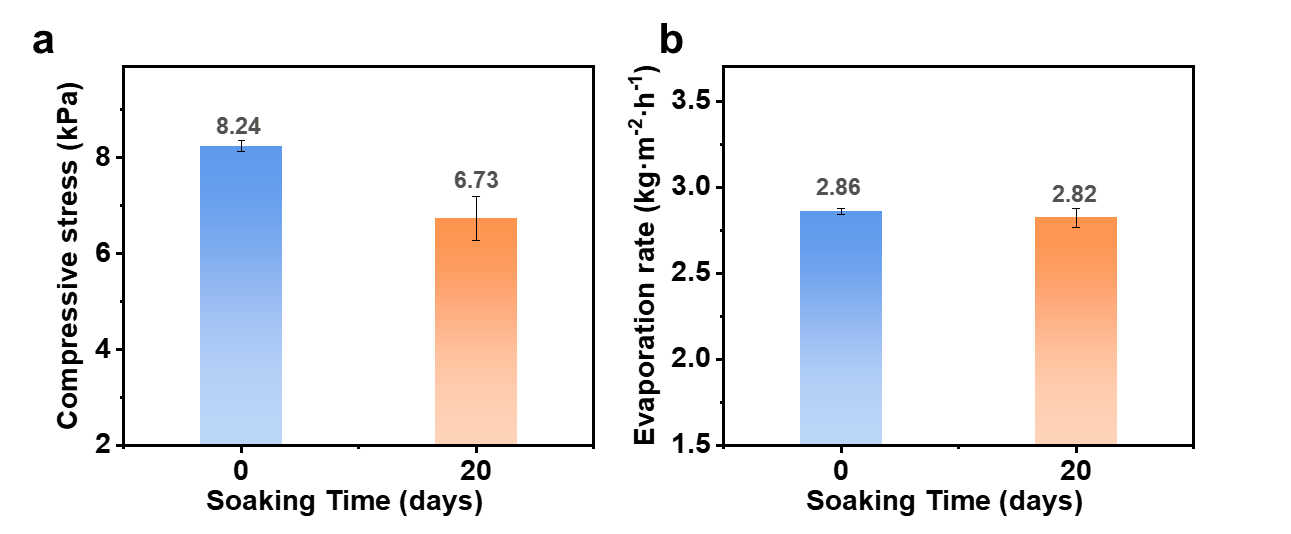
**

**Fig. S23** Stability comparison of the CA37-CB aerogel before and after soaking in natural seawater for 20 days. Wet-state compressive stress (**a**) and evaporation rate (**b**).

To further evaluate the long-term stability of the CA37-CB aerogel in a practical marine environment, the sample was soaked in natural seawater under outdoor conditions for 20 days. As shown in Fig. S22, the seawater-soaked CA37-CB aerogel still exhibited excellent compression recovery. After transverse pressing, the aerogel rapidly recovered its original shape within 1 s after release, indicating that the porous network was not destroyed during long-term seawater exposure. Axial cyclic compression tests further confirmed its wet-state mechanical stability, as the compression curves nearly overlapped during repeated loading and unloading. The wet-state compressive stress after 20 days of soaking remained 6.73 kPa, close to the original value of 8.24 kPa (Fig. S23a). In addition, the evaporation performance was almost unchanged after seawater soaking. The soaked CA37-CB aerogel maintained a high evaporation rate of 2.82 kg m^-2^ h^-1^, comparable to that of the original sample (2.86 kg m^-2^ h^-1^) (Fig. S23b). These results demonstrate that although CA37-CB is composed of biodegradable natural polysaccharides, it does not disintegrate or lose functionality during seawater operation, confirming its practical durability for long-term solar desalination.

**Note S1: Characterization and Solar-driven interfacial evaporation experiment**

**1. Characterization**

The surface morphology and microstructure of the photothermal materials and aerogel samples were observed with field emission scanning electron microscopy (FE-SEM, JSM-IT800SHL, Japan). The hydrophilicity of the aerogels was evaluated with a water contact angle meter (DM400, Kyowa, Japan). The chemical composition of the aerogel samples was analyzed by Fourier transform infrared spectroscopy (FTIR-6600, JASCO, Japan). Raman spectroscopy (NRS-3100, JASCO, Japan) was employed to characterize the photothermal material and to examine the water molecular states in the wet aerogels. The evaporation enthalpy of pure water and wet aerogel samples was measured by a differential scanning calorimeter (DSC 214 Polyma, NETZSCH, Germany). The light absorption of the aerogel evaporators was recorded with a UV-VIS-NIR spectrophotometer (UV-3600, Shimadzu, Japan). The compression properties of the biomass aerogels were tested by a universal testing machine (MCT-2150, A&D, Japan).

**2. Solar-driven interfacial evaporation experiment**

Simulated sunlight was provided by a solar simulator equipped with an AM 1.5G filter (XES-40S3-TT, SAN-EI Electric, Japan). The evaporation system was placed on an electronic balance (FX-200GD, A&D, Japan), and the mass was recorded every minute. The surface temperature of the aerogel evaporator was monitored using a thermocouple (NR-TH08, Keyence, Japan) and an infrared thermal imager (FLIR ONE PRO, FLIR, USA). The formation of the surface water film and the salt crystallization behavior were captured with a high-speed microscope (VW-9000, Keyence, Japan). Metal ion concentrations before and after evaporation were analyzed using an inductively coupled plasma optical emission spectrometer (ICP-OES, SPS3100, Hitachi, Japan). Seawater salinity was measured with a salinometer (DSM-25, LICHEN Instrument Technology, China). The absorbance of dye solutions was scanned using a UV-VIS spectrophotometer (UV-2700, Shimadzu, Japan). The electrical resistance of seawater and the extended fish-gill-inspired thermoelectric monitoring test were performed with a digital multimeter (DMM6500, Keithley, USA). All experiments were carried out in a controlled laboratory environment at 25 °C and 45% relative humidity.

**Note S2: Multiphysics Simulation of Heat–Flow–Salt Coupled Transport in the Porous Aerogel Evaporator**

**1. Model Overview**

This work establishes a three-field coupled numerical model using COMSOL Multiphysics (Version 5.3) to investigate heat conduction, water transport, and salt accumulation within a porous aerogel evaporator under solar illumination. The aerogel is treated as an isotropic porous medium, with its porosity, permeability, and effective thermal conductivity derived from experimental measurements or literature data. The model incorporates three physics interfaces: Laminar Flow in Porous Media (Brinkman–Darcy), Heat Transfer in Porous Media, and Transport of Diluted Species. By coupling the convective transport of temperature and salinity by the flow field, the temperature dependence of material properties, and interfacial cooling and salt enrichment induced by evaporation, this model can effectively replicate the temperature distribution, water replenishment pathways, and surface salt accumulation trends observed in practical evaporators.

**2. Governing Equations**

**Fluid Flow in Porous Medium (Brinkman–Darcy Model)**

$\nabla\cdot(\rho u)=0$ (S1)

$\rho(u\cdot\nabla)u=-\nabla p+\mu_{\text{eff}}\nabla^{2}u-\frac{\mu}{k}u-\rho g$ (S2)

Where $\rho$ is the water density, $u$ is the Darcy velocity field, $p$ is the pressure, $\mu$ and $\mu_{\text{eff}}$ are the dynamic and effective viscosities, respectively, $k$ is the permeability, and $g$ is the gravitational acceleration vector.

**Heat Transfer in Porous Medium**

The overall volumetric heat capacity of the porous medium is given by:

$\rho C_{p,\text{tot}}=\varepsilon\rho_{w}C_{p,w}+(1-\varepsilon)\rho_{s}C_{p,s}$ (S3)

Where $C_{p,\text{tot}}$ is the equivalent volumetric heat capacity, $\varepsilon$ is the porosity, $\rho_{w}$ and $\rho_{s}$ are the densities of water and the solid skeleton, respectively, and $C_{p,w}$ and $C_{p,s}$ are their specific heat capacities.

The heat conduction process is governed by:

$\rho C_{p,\text{tot}}(u\cdot\nabla T)-\nabla\cdot(k_{\text{eff}}\nabla T)=Q+Q_{\text{evap}}$ (S4)

Where $k_{\text{eff}}$ is the effective thermal conductivity, $T$ is the temperature, $Q$ is the volumetric heat source (e.g., solar energy deposition), and $Q_{\text{evap}}$ is the latent heat loss term due to evaporation.

**Salt Transport (Convection–Diffusion Equation)**

The salt convection-diffusion equation is:

$\frac{\partial c}{\partial t}+u\cdot\nabla c=\nabla\cdot(D_{\text{eff}}\nabla c)+S_{\text{evap}}$ (S5)

Where $c$ is the salt concentration, $D_{\text{eff}}$ is the effective diffusion coefficient, and $S_{\text{evap}}$ is the source term accounting for salt enrichment due to evaporation.

The salt flux at the evaporating surface is:

$-n\cdot(-D_{\text{eff}}\nabla c+cu)=J_{\text{evap}}(x,y)$ (S6)

Where $n$ is the outward unit normal vector, and $J_{\text{evap}}(x,y)$ is the applied evaporative mass flux.

The evaporative flux is formulated as:

$J_{\text{evap}}(x,y)=J_{0}\left[ 1+0.1\cdot\text{an1}(x,y) \right]$ (S7)

Where $J_{0}$ is the average evaporative flux, and $\text{an1}(x,y)$ is a random perturbation function in COMSOL, used to simulate spatial fluctuations in evaporation, making the model more representative of a real evaporating interface.

**3. Boundary Conditions**

**Thermal Boundaries**

Top Evaporating Surface

Solar heat flux:

$Q=\alpha q_{i}=0.98\times1000=980\text{ }\text{W m}^{-2}$ (S8)

Convective heat transfer boundary:

$-k_{\text{eff}}\nabla T\cdot n=h(T_{0}-T)$ (S9)

Side Walls (Adiabatic):

$-k_{\text{eff}}\nabla T\cdot n=0$ (S10)

Bottom (Fixed Temperature):

$T=T_{0}=291.15\text{ K}$ (S11)

**Flow Field Boundaries**

Top Free Outlet:

$p=p_{0}, u\cdot n=0$ (S12)

Side Walls (Impermeable):

$u\cdot n=0$ (S13)

Bottom (No-slip):

$u=0$ (S14)

**Salinity Boundaries**

Top Evaporating Surface:

$-n\cdot(-D_{\text{eff}}\nabla c+cu)=J_{\text{evap}}(x,y)$ (S15)

Bottom Reservoir:

$c=c_{0}$ (S16)

Side Walls (No Salt Flux):

$-n\cdot J=0$ (S17)

**4. Material and Model Parameters**

**Table S1** Aerogel Material Parameters

| **Parameter category** | **Parameter** | **Symbol** | **Value** | **Unit** | **Physical meaning / use** |
| --- | --- | --- | --- | --- | --- |
| Porous-medium parameter | Porosity | ε | 0.9 | — | Pore-volume fraction of the CA37-CB porous evaporator |
| Porous-medium parameter | Permeability | k | 8×10^−9^ | m^2^ | Water permeability of the porous evaporator |
| Heat-transfer parameter | Effective thermal conductivity | k_eff_ | 0.6 | W·m^-1^·K^-1^ | Effective heat conduction through the porous evaporator |
| Mass-transfer parameter | Diluted-species diffusion coefficient | D_eff_ | 4 × 10^-10^ | m^2^·s^-1^ | Diffusion of salt/solute in the porous evaporator |
| Photothermal parameter | Solar absorptance | α | 0.98 | — | Fraction of incident solar energy absorbed by the evaporator |
| Heat-transfer parameter | Convective heat-transfer coefficient | h | 10 | W·m^-2^·K^-1^ | Heat exchange between the evaporator and ambient air |
| Environmental parameter | Ambient temperature | T_amb_ | 25 | °C | Experimental ambient temperature |
| Environmental parameter | Relative humidity | RH | 45 | % | Experimental relative humidity |
| Model parameter | Initial temperature | T₀ | Measured initial temperature | °C | Initial thermal condition used in the simulation |
| Fluid parameter | Water density | ρ_w_ | 1000 | kg·m^-3^ | Water property used for water transport and equivalent flux conversion |
| Fluid parameter | Water dynamic viscosity | μ | 1.0 × 10^-3^ | Pa·s | Fluid property used in the flow / Brinkman-Darcy model |
| Mass-transfer parameter | Initial salt concentration | c₀ | Experimental saline solution | mol·m^-3^ | Initial concentration for salt transport |

**Table S2** Model contrast between the CA37-CB-F and CA37-CB-SF configurations

| **Physics field** | **Boundary / parameter** | **CA37-CB-F model** | **CA37-CB-SF model** | **Physical meaning** |
| --- | --- | --- | --- | --- |
| Flow field | Bottom water-supply boundary | Restricted foam-mediated water supply, u_F_ | Direct water-contact supply, u_SF_ | Main difference in water-supply mode |
| Flow field | Water transport rate | Lower, u_F_ | Higher, u_SF_ > u_F_ | Direct water contact provides more sufficient water replenishment |
| Diluted species transport | Effective transport region | Restricted effective transport region | Larger effective transport region | More water/salt transport occurs in CA37-CB-SF |
| Diluted species transport | Convective solute transport | Lower, u_F_ c | Higher, u_SF_ c | Faster water flow enhances solute/salt convection |
| Salt transport | Top surface | Evaporation-induced salt enrichment | Evaporation-induced salt enrichment | Same evaporation-driven salt-enrichment mechanism |
| Salt transport | Side wall | No salt flux | No salt flux | Same lateral salt boundary |

**5. Geometry and Mesh**

The model dimensions are 2.5 cm × 2.5 cm in plane, with a thickness of approximately 1 cm. The mesh consists of tetrahedral elements, with local refinement applied at the evaporating surface to resolve gradients in temperature, velocity, and salinity.

**6. Mesh Independence and Sensitivity Analysis**

To verify mesh independence, the global mesh size was reduced by 25%. The resulting changes in temperature, velocity, and salinity distributions were all less than 3%, indicating sufficient mesh resolution. A sensitivity analysis was performed by perturbing key parameters by ±10%. The results showed changes in the temperature and salinity fields of less than 5%, demonstrating the robustness of the model.

**7. Model Validation**

The simulation exhibits a temperature distribution characterized by a warmer evaporating surface and a cooler bottom region, which matches well with the infrared thermal imaging results obtained in experiments. Meanwhile, the model successfully reproduces the upward water replenishment driven by the capillary channels inside the aerogel, consistent with the observed liquid transport behavior. In addition, the progressive accumulation of salt at the air–water interface is clearly captured in the simulation, reflecting the same crystallization tendency recorded during high-salinity evaporation tests. The agreement between these key physical phenomena and experimental observations supports the reliability and validity of the established numerical model.

**Note S3 Cytotoxicity test of biomass CA37**-**CB aerogel**

NIH 3T3 fibroblasts were maintained at 37 °C in a humidified atmosphere containing 5% CO_2_, using complete RPMI-1640 medium supplemented with 10% fetal bovine serum (FBS) and 1% penicillin–streptomycin. To prepare the sample extract, CA37-CB aerogels were immersed in serum-free RPMI-1640 at a mass-to-volume ratio of 10 mg mL^–1^ and incubated for 24 h at 37 °C. The resulting extract was sterilized by passing through a 0.22 μm filter and then supplemented with 10% FBS and 1% penicillin–streptomycin. Complete RPMI-1640 medium was used as the blank control. NIH 3T3 cells were seeded in 96-well plates at 1000 cells per well and allowed to adhere overnight before exposure to the different media. Cell viability after 24 h and 48 h was quantified using the CCK-8 assay (Beyotime, China). In parallel, cells treated under the same conditions were subjected to live/dead staining (Beyotime, China), and fluorescence images were collected using an inverted fluorescence microscope to visualize cellular morphology and viability.

**Note S4 Calculation method and interpretation of apparent solar-to-vapor efficiency**

The solar steam evaporation efficiency was calculated using the temperature-dependent apparent efficiency method, which considers both the latent heat of water evaporation and the sensible heat required for increasing the water temperature. The calculated efficiency higher than 100% should be regarded as an apparent solar-to-vapor efficiency. Similar values have been reported in hydrogel and polymer-network-based solar evaporators, where the water state is regulated and the effective evaporation energy is reduced. Therefore, this value is mainly used for comparison under the same calculation method, while the evaporation rate and long-term salt resistance are also emphasized as key performance indicators.
